# Stable transgenic C9orf72 zebrafish model key aspects of the ALS/FTD phenotype and reveal novel pathological features

**DOI:** 10.1186/s40478-018-0629-7

**Published:** 2018-11-19

**Authors:** Matthew P. Shaw, Adrian Higginbottom, Alexander McGown, Lydia M. Castelli, Evlyn James, Guillaume M. Hautbergue, Pamela J. Shaw, Tennore M. Ramesh

**Affiliations:** 1 Sheffield Institute for Translational Neuroscience, University of Sheffield, 385a Glossop Road, Sheffield, S10 2HQ UK; 2 The Bateson Centre, Firth Court, The University of Sheffield, Western Bank, Sheffield, S10 2TN UK

**Keywords:** Amyotrophic lateral sclerosis, C9orf72, SOD1, TDP-43, Zebrafish, Drug-screening

## Abstract

A hexanucleotide repeat expansion (HRE) within the chromosome 9 open reading frame 72 (*C9orf72*) gene is the most prevalent cause of amyotrophic lateral sclerosis/fronto-temporal dementia (ALS/FTD). Current evidence suggests HREs induce neurodegeneration through accumulation of RNA foci and/or dipeptide repeat proteins (DPR). *C9orf72* patients are known to have transactive response DNA binding protein 43 kDa (TDP-43) proteinopathy, but whether there is further cross over between *C9orf72* pathology and the pathology of other ALS sub-types has yet to be revealed.

To address this, we generated and characterised two zebrafish lines expressing *C9orf72* HREs. We also characterised pathology in human *C9orf72*-ALS cases. In addition, we utilised a reporter construct that expresses DsRed under the control of a heat shock promoter, to screen for potential therapeutic compounds.

Both zebrafish lines showed accumulation of RNA foci and DPR. Our C9-ALS/FTD zebrafish model is the first to recapitulate the motor deficits, cognitive impairment, muscle atrophy, motor neuron loss and mortality in early adulthood observed in human *C9orf72*-ALS/FTD. Furthermore, we identified that in zebrafish, human cell lines and human post-mortem tissue, *C9orf72* expansions activate the heat shock response (HSR). Additionally, HSR activation correlated with disease progression in our C9-ALS/FTD zebrafish model. Lastly, we identified that the compound ivermectin, as well as riluzole, reduced HSR activation in both C9-ALS/FTD and SOD1 zebrafish models.

Thus, our C9-ALS/FTD zebrafish model is a stable transgenic model which recapitulates key features of human *C9orf72*-ALS/FTD, and represents a powerful drug-discovery tool.

**Electronic supplementary material:**

The online version of this article (10.1186/s40478-018-0629-7) contains supplementary material, which is available to authorized users.

## Introduction

Amyotrophic lateral sclerosis (ALS) is a neurodegenerative disorder characterised by motor neuron loss, leading to progressive muscle weakness and eventual death, primarily due to respiratory failure. Approximately 10% of ALS is inherited in an autosomal dominant fashion, this is known as familial-ALS (fALS). The remaining 90% of ALS cases are caused by complex genetic and environmental interactions which are currently not well understood, this is known as sporadic-ALS (sALS). Mutations in multiple genetic loci have been identified as causes of ALS including the *SOD1* and *TARDBP* loci. See Amyotrophic Lateral Sclerosis Online Genetics Database for comprehensive information (http://alsod.iop.kcl.ac.uk/). The most common known genetic cause of ALS and frontotemporal dementia (FTD) is a hexanucleotide expansion within the first intron of the *C9orf72* gene [11, 32]. Carriers of the *C9orf72* hexanucleotide expansion may show symptoms of ALS or FTD exclusively, but can also present with symptoms of both diseases concurrently.

Concerning pathology in *C9orf72* patients, there are three major, non-mutually exclusive routes of toxicity which have been proposed to arise from the *C9orf72* expansion: **1)** Sense and antisense RNA foci which sequester RNA binding proteins causing dysregulation of RNA processing [7, 11]. **2)** Dipeptide repeat proteins (DPRs) produced via non-canonical repeat associated non-ATG (RAN) translation, form insoluble aggregates in the nucleus and cytoplasm [42]. **3)** Hexanucleotide expansion mediated haploinsufficiency may cause dysregulation of endogenous C9orf72 pathways such as autophagy [12, 39].

To date, several models have been generated to help dissect out the mechanisms of *C9orf72* expansion mediated toxicity. Most drosophila and zebrafish models support an RNA/DPR mediated gain of toxic function hypothesis [18, 26, 28, 37]. In addition, transgenic mouse models have been generated containing the human patient *C9orf72* gene (complete with G_4_C_2_ expansion and flanking regions). Two transgenic mouse models demonstrate the reduced survival, neuronal loss and motor deficits observed in human C9-ALS/FTD [14, 20]. However, a further two independently generated *C9orf72* transgenic models showed no signs of neuronal loss or reduced survival. [29, 30]. This highlights the wide variability observed in *C9orf72* expansion in vivo models*. C9orf72* knockdown in the zebrafish causes mild motor defects [6]. However, early reports from *C9orf72* knockout zebrafish do not recapitulate the knockdown motor phenotypes ([34]; Schmid, Hruscha, Haass, unpublished). Additionally, four independently generated *C9orf72* knockout mice did not demonstrate any neurodegenerative phenotype [1, 13, 17, 35].

Whilst mouse models are a useful tool for understanding the pathobiology of *C9orf72*-related ALS, they are not amenable to high throughput drug screening. Genetic modifier screens have been carried out in drosophila, but their CNS is much simpler compared to the human CNS and findings in this invertebrate model are less likely to translate to the clinic [3, 15]. Zebrafish are vertebrates with a more complex CNS, and therefore represent a practical compromise for assessing the efficacy of therapeutic compounds.

Here we present a novel transgenic zebrafish model which stably expresses *C9orf72* expansions. These zebrafish recapitulate the behavioural deficits, cognitive abnormalities, motor decline and early mortality observed in C9-ALS patients. Additionally we show that *C9orf72* expansions activate the heat shock response in human cell lines, post-mortem ALS tissue and our model zebrafish. Using these *C9orf72* zebrafish and our previously reported *SOD1* zebrafish in tandem [31], we show that riluzole and a newly identified compound, ivermectin, are able to reduce cellular stress in both *C9orf72* and *SOD1* in vivo models. We therefore propose that our *C9orf72* zebrafish model effectively bridges the gap between drosophila and mouse models by providing an efficient tool for high-throughput in vivo drug screening assays.

## Materials and methods

### Generating and maintenance of transgenic zebrafish

**Fig. 1:**
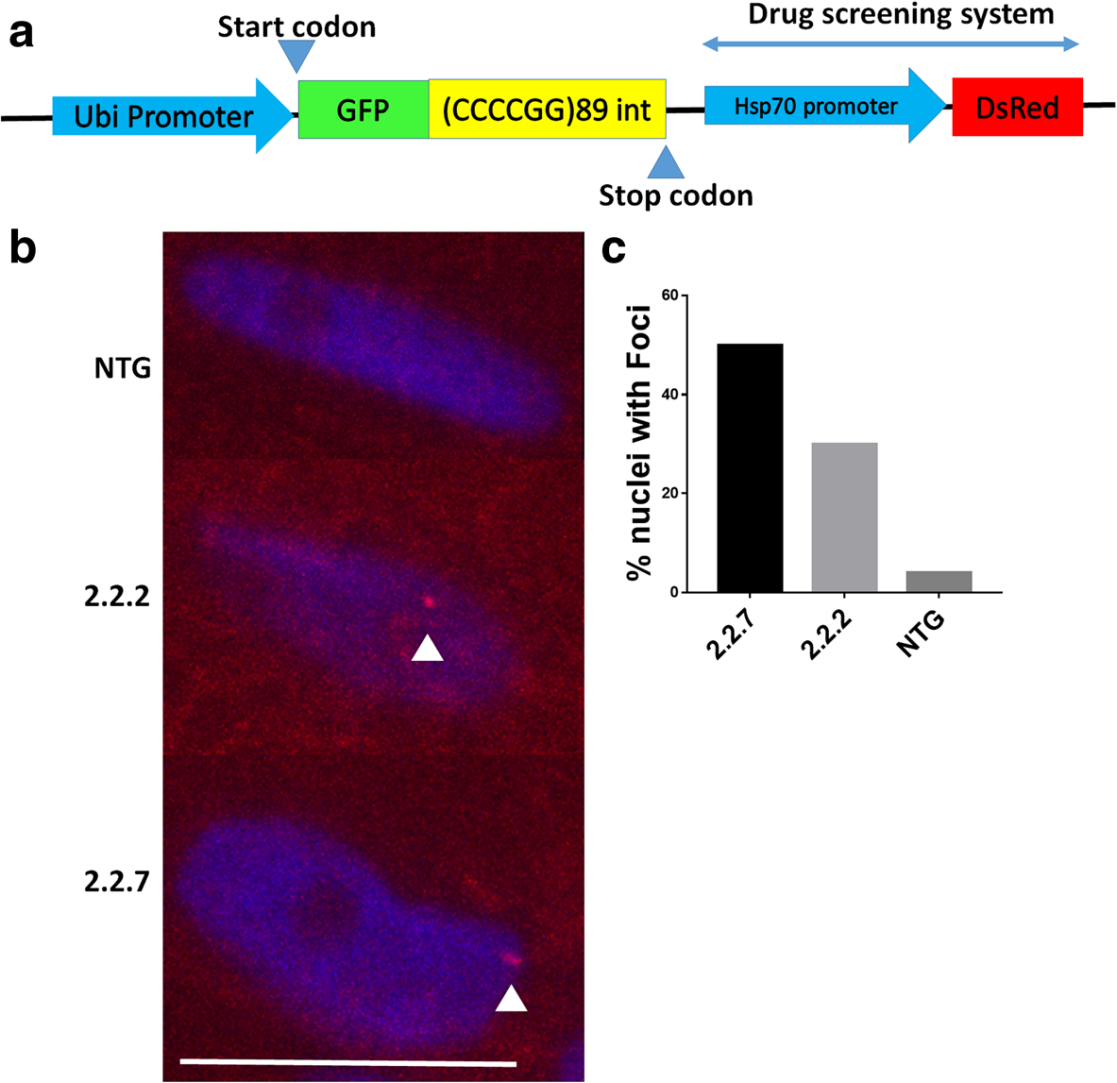
*C9orf72* model zebrafish display RNA foci in the nucleus. (**a**) Schematic representation of the transgene inserted into 2.2 zebrafish. A zebrafish ubiquitin promotor drives GFP-DPR expression. An *hsp70* promotor then drives DsRed production as a read out of cellular stress. (**b**) In situ hybridisation of paraffin embedded sections of 10dpf 2.2–7 zebrafish using a Cy3-conjugated (red) GC probe (GGGGCC)X4 showed that RNA foci are present in the nuclei of muscle cells. Arrow heads denote RNA foci. Scale bar = 10 μm. (**c**) Quantification of RNA foci showed Immunofluorescence labelling of adult zebrafish muscle tissue showed that poly-GP DPR protein localises to the nucleus in 2.2–2 and 2.2–7 transgenic zebrafish

Zebrafish embryos were injected with a DNA construct containing 89 C9orf72 hexanucleotide repeats driven by a zebrafish ubiquitin promotor (Fig. 1a, Additional file 1). Creation and identification of transgenic zebrafish was performed as previously described [31] and maintained using established practices [40].

### In situ hybridisation and immunofluorescence

In situ hybridization of paraffin embedded tissue sections to detect CCCCGG (C_4_G_2_) foci was performed on 5dpf embryos using methods described previously [8]. For immunofluorescence staining, paraffin embedded tissue was dewaxed, antigen retrieved and stained as previously described [9].

### Western blotting

Ethical approval for use of human cerebellum samples was obtained by the Sheffield Brain Tissue Bank Management Board, and approval to release tissue under REC 08/MRE00/103 was granted. Human cerebellum samples and adult zebrafish tissue, brain, spinal cord and whole zebrafish embryos were snap frozen in liquid nitrogen and processed for western blotting. Laemmli buffer was added in the ratio of 10 μl:1 mg of tissue and sonicated. SDS-PAGE and immunoblotting were performed as previously described [39]. Antibodies used were Rb-anti-PR (gift from Dieter Edbauer), Rb-anti-Dsred (Clontech 632,496), Ms-anti-tubulin (Abcam). Species specific HRP conjugated secondary antibodies were used and imaged by chemiluminescence using G-Box.

### Embryonic behaviour

For spontaneous locomotor activity, 5 dpf zebrafish were placed into individual wells of a 96well plate and habituated in the dark for 10 min before a light stimulus was turned on. 10 min of light was followed by 10 min dark and repeated once more. Recordings were carried out using ZebraBox software (ViewPoint Behaviour Technologies), movement thresholds used were slow (x < 5 mm/sec), intermediate (5 < x < 15 mm/sec) and fast (x > 15 mm/sec).

For centre avoidance behaviour, 5 dpf zebrafish were placed into a 6 well plate at a density of 30 zebrafish per well. After a 30 min habituation period with the lights on, the lights were turned off for 5 min then on for 5 min for 6 cycles. Frame grab was performed at 30 s for every minute in the lights on condition using the Imagegrab tool, and this was repeated for each of the 6 lights on periods. Using ImageJ, circles of the same size were placed around the outside of every well so that only the centre of the well was visible, the % of zebrafish present in the centre of the well was then blind counted for every image and the average per well was calculated.

### Adult locomotor behaviour

Zebrafish swimming ability was tested using a swim tunnel with an intial flow-rate of 2 L/min, increasing in 2 L/min increments every 5 min until the maximum flow rate of 11.6 L/min was achieved. Data were analysed as previously described [31]. 5 min post-testing, the spontaneous swimming behaviour of the fish was measured for 30 min using a camera linked to ZebraLab software (ViewPoint Behaviour Technologies). Speed thresholds used were slow (x < 60 mm/sec), intermediate (60 < x < 120 mm/sec) and fast (x > 120 mm/sec).

### Motor neuron counts and myotome measurements

Spinal motor neurons were counted from paraffin embedded adult zebrafish segments cut anterior to the pelvic fin, sectioned at 10 μm and stained with haematoxylin and eosin. Cells with a soma size >75μm^2^ and within 25,000μm^2^ proximity of the central canal were designated as motor neurons. Three sections/per animal were analysed by two independent blinded investigators and averaged. The areas of individual myotomes were measured by a blinded investigator from 6 images per animal. All muscle images were obtained from the epaxial muscle region just lateral to the dorsal spinal bone. Any myotome which was incomplete due to being partially out of frame was not included in the analysis.

### Cell culture and transfections

Cells were maintained in a 37 °C incubator with 5% CO2. HEK293T cells were cultured in Dulbecco’s Modified Eagle Medium (Sigma) supplemented with 10% foetal bovine serum (FBS) (Gibco) and 5 U ml^− 1^ Penstrep (Lonza). Neuro-2a(N2A) (ATCC) cells were cultured in Dulbecco’s Modified Eagle Medium (Sigma) supplemented with 10% FBS (Gibco), 5 U ml^− 1^ Penstrep (Lonza) and 5 mM sodium pyruvate.

HEK293T and N2A cells were transfected with 700 ng of plasmid using 3.5 μg PEI/ml media and one tenth media volume of OptiMEM in a 24 well format. Approximately, 50,000 HEK293T cells were seeded / well and 75,000 N2A cells were seeded per well of the 24 well plate*. Proteins were extracted 72 h post-transfection. Cells were washed in ice cold phosphate buffered saline (PBS) and subsequently lysed in ice cold lysis buffer (*50 mM Hepes pH 7.5, 150 mM NaCl, 10% glycerol, 0.5% Triton X-100, 1 mM EDTA, 1 mM DTT, protease inhibitor cocktail (Sigma)) for 10 min on ice. Extracts were then centrifuged at 17,000 *g* for 5 min at 4 °C. Extracts were quantified using Bradford Reagent (BioRAD), resolved by SDS-PAGE, electroblotted onto nitrocellulose membrane and probed to the relevant primary antibodies.

### Heat shock cell stress drug screening assay

At 2 dpf, transgenic zebrafish were placed into a 96 well plate in 200 μl of drug or DMSO containing E3 zebrafish media. At 5 dpf zebrafish were sonicated in the well for 10 s each and then centrifuged in a plate spinner at 3000 rpm for 10 min. From each well, 20 μl of supernatant was transferred into a 385 well plate, and the DsRed levels in each individual lysate were quantified using a FLUOstar Omega fluorescence plate reader (BMG labtech).

## Quantification and statistical analysis

Data were analysed by one way ANOVA with Tukey’s post hoc test or two way ANOVA with Sidak’s post hoc test for multiple comparisons, t-test or Kaplan Meier analysis as indicated in the appropriate figure legend. Significance is denoted as * *P* < 0.05, ** *P* < 0.01, *** *P* < 0.001 and **** *P* < 0.0001. Individual myotome size data were counted into bins with a 0.5mm^2^ size range. The frequency distribution of each genotype was then compared using a chi-squared test for trend.

## Results

### Generation of transgenic zebrafish

To better understand ALS/FTD pathogenesis and screen potential therapeutic agents, we generated a *C9orf72* zebrafish model. At the single cell stage zebrafish embryos were injected with a DNA construct containing 89 C9orf72 hexanucleotide repeats (Fig. 1a, Additional file 1). Of the 3 zebrafish lines generated, one was extremely toxic, resulting in death within 7 days of fertilisation (dpf). Therefore, only the 2 remaining lines were maintained to breeding age and established for further characterisation. These two transgenic zebrafish lines which were established to adulthood will henceforth be known as line 2.2–2 and line 2.2–7, or collectively as 2.2-zebrafish lines. Both 2.2-zebrafish lines give rise to 1:1 ratios of transgenic:NTG offspring when outbred, suggesting a single site of transgene insertion.

### *C9orf72* zebrafish lines express RNA foci and DPR

**Fig. 2:**
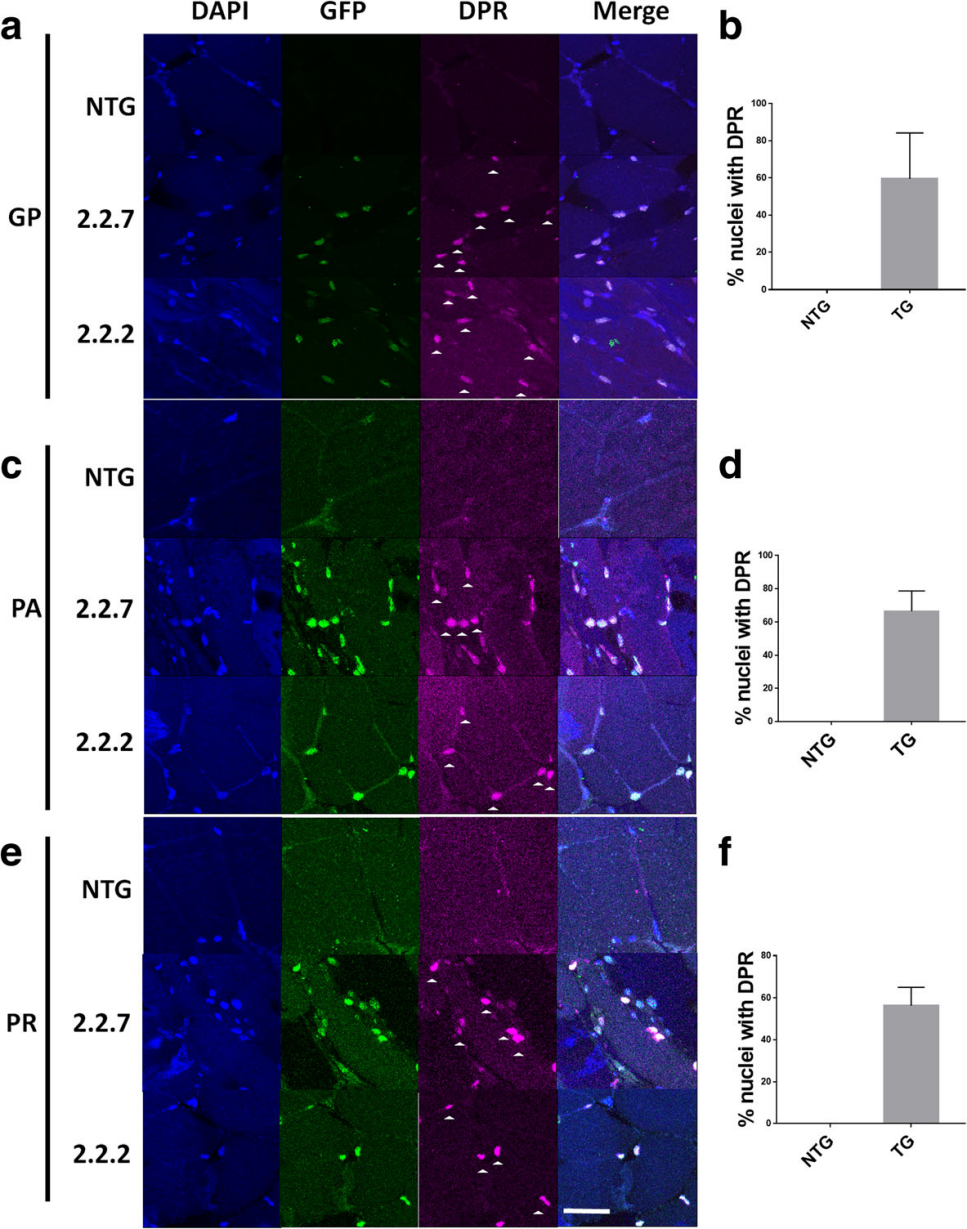
C9orf72 model zebrafish display DPR expression in the nucleus. (**a,b**) Immunofluorescence labelling of adult zebrafish muscle tissue showed that poly-GP DPR protein localises to the nucleus in 2.2–2 and 2.2–7 transgenic zebrafish. (**c.d**) Immunofluorescence labelling of adult zebrafish muscle tissue showed that poly-PA DPR protein localises to the nucleus in 2.2–2 and 2.2–7 transgenic zebrafish. (**e,f**) Immunofluorescence labelling of adult zebrafish muscle tissue showed that poly-PA DPR protein localises to the nucleus in 2.2–2 and 2.2–7 transgenic zebrafish. For all DPR images nuclei are stained with Hoechst (blue), GFP is stained with GFP antibody (green) and DPR proteins are stained with the relevant DPR antibody (purple), white arrow heads denote DPR positive staining. Scale bar = 25 μm for all DPR images

The hallmark features of *C9orf72* pathology are expression of RNA foci and DPR species. Using in situ hybridisation and immunofluorescence, we identified expression of RNA foci and DPR species in both 2.2-zebrafish lines. Antisense RNA foci (CCCCGG, the same orientation with respect to the construct) can be detected in the nuclei of muscle cells in both 2.2-zebrafish lines (Fig. 1b), no more than one focus is observed per nucleus, and no cytoplasmic foci were detected. 50% (11/22) of nuclei in 2.2.7 line showed RNA nuclear foci while fewer foci (30%, 6/20) were observed in 2.2.2 line. Non-transgenics showed 4% (1/25) foci like staining but failed to show colocalisation in the nuclei (Fig. 1c). It is presumed, that the single focus observed in the NTG zebrafish was due to non-specific binding of the in situ probe. To determine whether repeat RNA was translated into DPR proteins, antibodies specific to antisense DPR species poly-GP, PA and PR were used. All three antisense DPR species were detected in the nuclei of muscle cells from both 2.2-zebrafish lines (Fig. 2a,c,e) with over 50% of the nuclei expressing the DPRs (Fig. 2b,d,f).

### *C9orf72* zebrafish produce multiple distinct DPR species

**Fig. 3:**
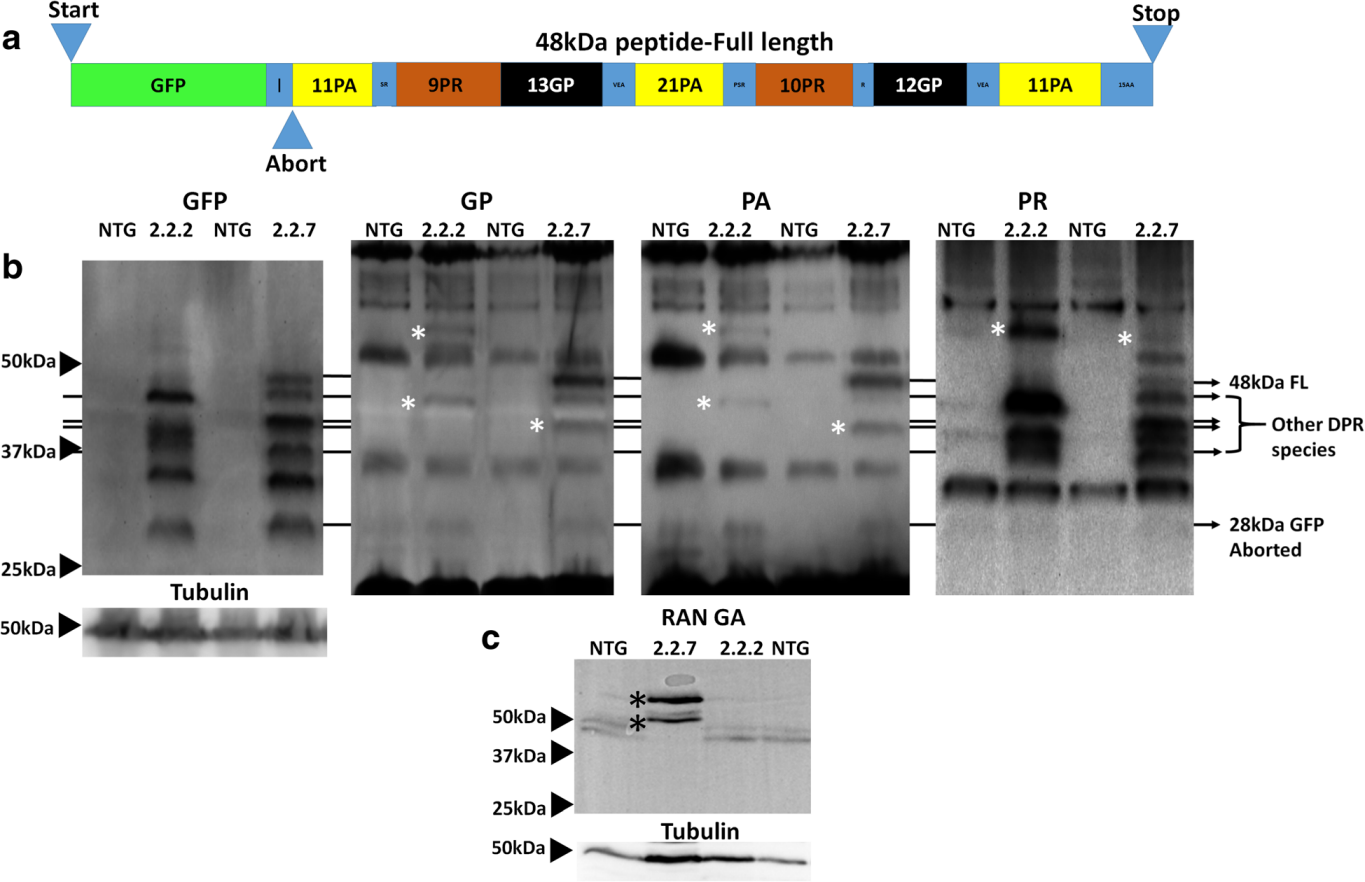
*C9orf72* model zebrafish produce multiple species of DPRs from sense and anti-sense transcripts (**a**) Schematic of the GFP-DPR fusion protein produced by AUG driven translation. (**b**) Anti-sense DPRs (predominately ATG-driven) containing full length-GFP-DPR fusion, intermediate GFP-DPR fusion and GFP alone bands are detected in 5dpf embryonic lysates. Non-AUG driven GFP deficient RAN translation DPR are also detected in both 2.2.2 and 2.2.7 transgenic lines (Asterix) (**C**) Sense Poly(GA) DPRs (exclusively RAN-translation driven) detected in 5dpf embryonic lysates

The various DPR species are known to have differential toxicity, with arginine rich species being considered the most toxic. To investigate whether there is a relationship between molecular weight (MW) and species toxicity, western blotting was performed on zebrafish lysates. The transgene construct expressed in both 2.2-zebrafish lines causes the production of GFP tagged DPR proteins via canonical ATG (start codon) dependent translation. The full length GFP fusion protein is predicted to be 48 kDa while any truncation 3′ of any C4G2 repeats would result in the production of GFP alone (28KDa). The schematic of the GFP-DPR fusion protein is shown (Fig. 3a). GFP tagged DPRs are produced from C_4_G_2_ transcripts and can be detected at 5 dpf (Fig. 3b, left most panel). Interestingly, multiple GFP bands are detected in both the 2.2–2 and 2.2–7 zebrafish lines, and these bands were often unique to one zebrafish line or the other, and were consistent over > 10 clutches. The differential expression of DPRs between 2.2–2 and 2.2–7 zebrafish also holds true when probing for the DPR proteins directly (Fig. 3b, 3 right panels). The full length GFP-DPR fusion at 48 kDa was expressed but at low levels **(**Fig. 3b, 48 kDa band)**.** Probing antibodies against DPR proteins also revealed that some DPR bands detected did not co-localise with any of the ATG-dependent translation bands detected using the GFP antibody, suggesting that these bands are likely to be produced via non-canonical RAN translation (Fig. 3b, three right panels marked with asterix).

In addition to the poly-PA, PR and GP DPRs produced from the (C_4_G_2_) RNA transcripts, we were also able to detect poly(GA) DPR produced from the (G_4_C_2_) RNA transcript, however poly(GA) was only detected in the 2.2–7 zebrafish line (Fig. 3c). The detection of poly(GA) indicates that bidirectional transcription of the GC rich region is occurring in the presence of our transgene. As the transcription of the RNA transcript containing the (G_4_C_2_) expansion is not driven by a conventional promotor region, this strongly indicates that poly(GA) protein is indeed produced via RAN translation.

**Fig. 4:**
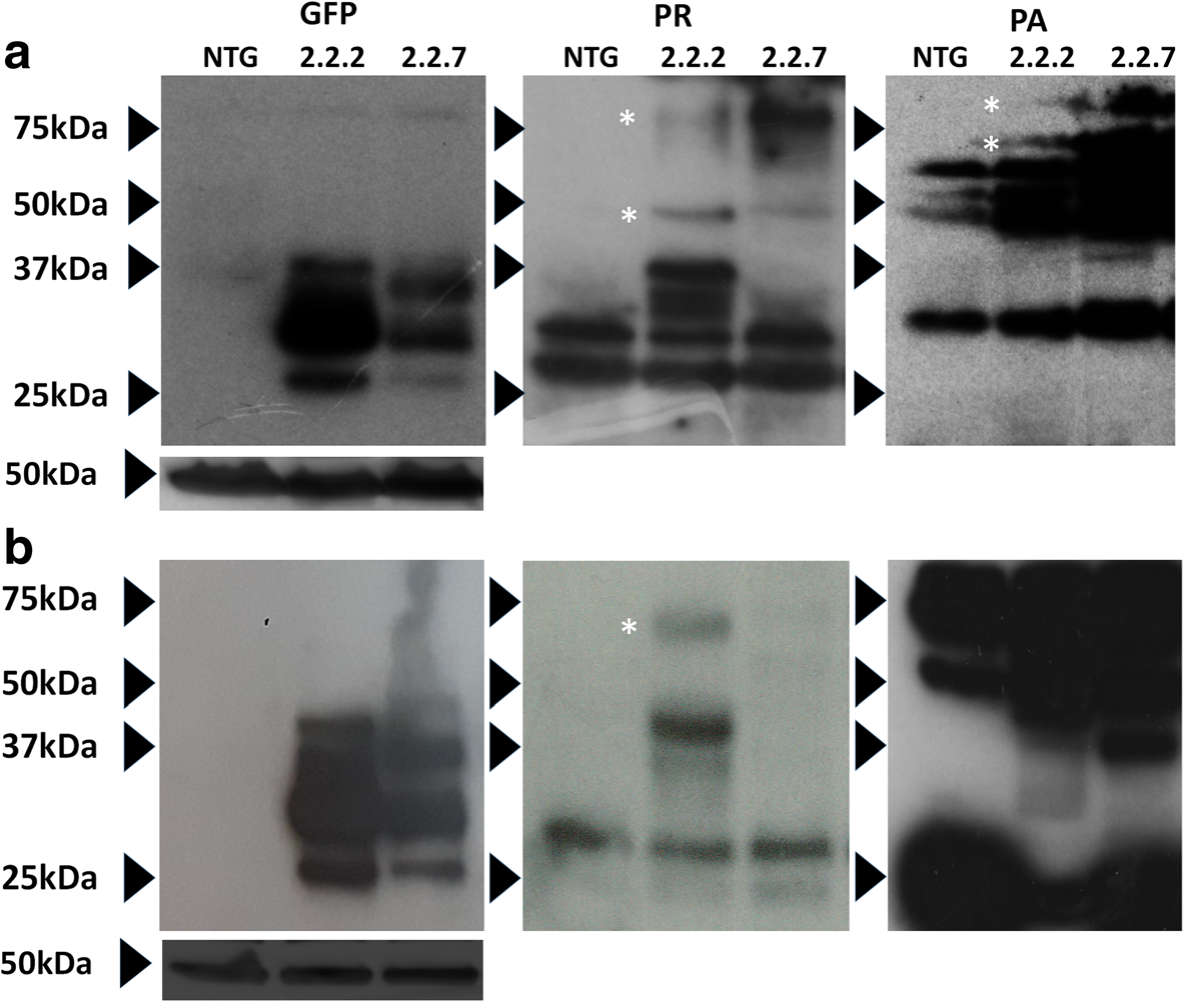
*C9orf72* model zebrafish produce multiple species of DPRs in the adult CNS. (**a**) Anti-sense DPRs (predominately ATG-driven) detected in adult brain lysates. (**b**) Anti-sense DPRs (predominately ATG-driven) detected in adult spinal cord lysates. Asterix (*) denotes protein bands which are proposed to have been produced via RAN-translation

In human ALS, *C9orf72* associated toxicity occurs primarily in cells of the CNS, and so it is essential to ascertain whether DPR species are also produced within the CNS of this *C9orf72* model zebrafish. In adult brain and spinal cord of both 2.2-zebrafish lines, GFP-tagged DPR species and DPR species which were not immunoreactive with GFP antibodies (RAN translation bands), could be detected (Fig. 4). This suggests that both ATG-dependent translation and RAN translation of DPR species occurs within the CNS of the 2.2-zebrafish.

In summary, both 2.2-zebrafish lines exhibit DPR species generated by conventional ATG-dependent translation and RAN translation in the muscle and the CNS. In addition, the 2.2–7 zebrafish line shows bi-directional transcription, producing DPR species from both G_4_C_2_ and C_4_G_2_ RNA transcripts. In both 2.2-zebrafish lines, the band pattern of DPRs detected largely remains constant from 5 dpf until adulthood, although higher MW (>50KDa) poly(PR) positive bands are more abundant in adult tissue. Of all the DPR species examined here, poly(PR) generally has the highest propensity to form high MW RAN-translation mediated bands.

### Early mortality, altered swimming behaviour and reduced weight gain in transgenic *C9orf72* zebrafish

**Fig. 5:**
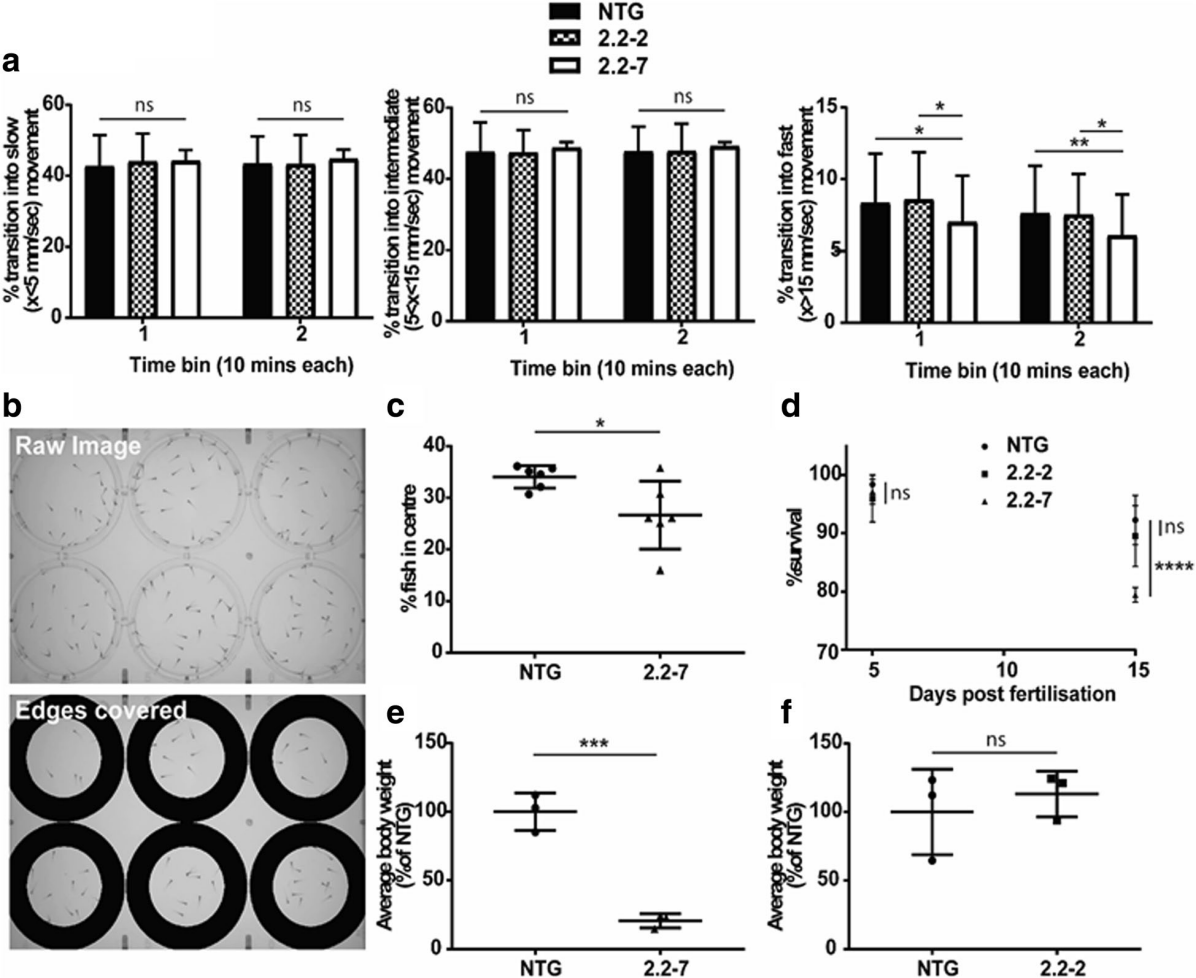
*C9orf72* model zebrafish show early motor deficits, behavioural defects and reduced viability. (**a**) When kept under dark conditions, no difference is observed in the proportion of times fish transition into a slow movement (left) or intermediate movement (middle). However, the proportion of transitions into fast movements is significantly reduced in 2.2–7 zebrafish. *N* = 60 individual fish per genotype, from 3 different clutches. (**b**) Representative images of the plate set-up used to monitor centre avoidance behaviour in zebrafish. 30 fish were placed in each well and one image every minute was analysed. Image shown as recorded (top) and then following removal of the region around the edge of the plate for analysis (bottom). In these images 2.2–7 are placed across the top 3 wells and NTG across the bottom 3 wells. (**c**) Quantification of centre avoidance behaviour showing 2.2–7 zebrafish are significantly less often found in the plate centre. N = 6 clutches per genotype. (**d**) Survival of zebrafish did not change by 5dpf, however by 15dpf survival of the 2.2–7 line was significantly reduced compared to NTG. *N* = 4 clutches per genotype. (**e**) At 30dpf 2.2–7 zebrafish have reduced average body weight in comparison to their NTG clutch mates. *N* = 3 clutches per genotype. (**f**) At 30dpf there was no difference in average body weight between 2.2–2 zebrafish and their NTG clutch mates. N = 3 clutches per genotype. All data are shown as mean +/− standard deviation; *P < 0.05, **P < 0.01, ***P < 0.001 and ****P < 0.0001

Neither 2.2-zebrafish lines showed any overt morphological abnormalities during embryonic development (0–5 dpf). At 5 dpf zebrafish begin to express a wider repertoire of behaviours, including more frequent swimming and independent feeding. For this reason, rigorous evaluation was performed on 5 dpf zebrafish to test for underlying motor and behavioural deficits. In order to test the spontaneous locomotor activity of embryonic zebrafish, we monitored 5 dpf zebrafish in 96 well plates using the Viewpoint behaviour monitoring setup. No significant difference was observed between the groups in the proportion of times transitioning occurred into slow or medium movements (Fig. 5a). However, a significant reduction in the proportion of transitions into fast movement was detected in 2.2–7 zebrafish, when compared to either NTG or 2.2–2 zebrafish (Fig. 5a).

As *C9orf72* expansions in human ALS cause a spectrum of both motor and cognitive deficits, we examined whether normal zebrafish behaviour was affected in 2.2–7 zebrafish at 5 dpf. Centre avoidance behaviour assays are a validated means of measuring willingness to explore in zebrafish [33], and are comparable to the open field test performed in mice. It was determined that 2.2–7 zebrafish were significantly less likely to venture into the centre of the well when compared to their NTG clutchmates (Fig. 5b+c).

To determine if the early embryonic expression of RNA foci and DPR impacted upon the viability of the *C9orf72* zebrafish, we carried out early (1–15 dpf) survival analysis. Heterozygous 2.2–2 zebrafish do not show any change in survival within 15 dpf as compared to NTG zebrafish (data from NTG clutchmates of all genotypes are pooled; Fig. 5d). However, heterozygous 2.2–7 zebrafish did show a significant decrease in survival within 15 dpf as compared to NTG zebrafish (Fig. 5d), but not in comparison to 2.2–2 zebrafish.

It was noted that during early development, the 2.2–7 zebrafish appeared smaller than their NTG clutchmates. At 30 dpf there was a significant decrease in total body weight of 2.2–7 zebrafish compared to their NTG clutchmates (Fig. 5e). However, 2.2–2 zebrafish did not show a significant difference in body weight as compared to their own clutchmates at the same age (Fig. 5f).

In summary, 2.2–7 zebrafish but not 2.2–2 zebrafish, show significant reduction in survival at 15 dpf, and reduction in bodyweight at 30 dpf. At 5 dpf, 2.2–7 zebrafish also show defects in swimming activity and displayed signs of atypical behaviour. Behaviour of the phenotypically more severe 2.2–7 zebrafish was also studied through adulthood.

### *C9orf72* zebrafish display adult onset ALS-like behavioural phenotypes

**Fig. 6:**
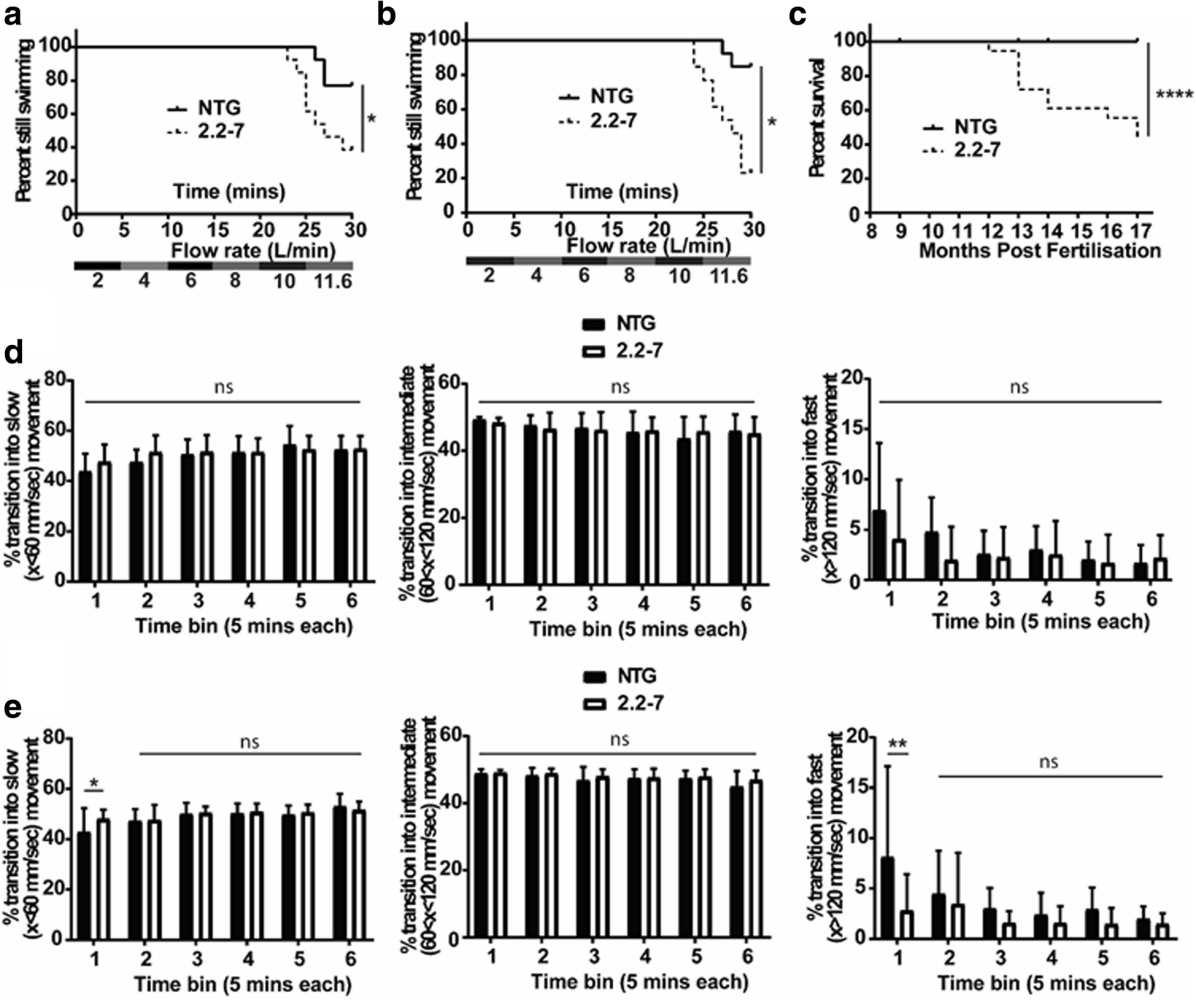
*C9orf72* model zebrafish show adult onset swimming endurance deficits and reduced survival. (**a**) At nine months old, 2.2–7 transgenic zebrafish failed to continue swimming at earlier time points than their NTG clutch mates. *N* = 13 fish per genotype. (**b**) Also at twelve months old, 2.2–7 transgenic zebrafish failed to continue swimming at earlier time points than their NTG clutch mates. N = 13 fish per genotype. (**c**) Adult transgenic 2.2–7 zebrafish have reduced survival between 8 and 17 months in comparison to their NTG clutch mates which are housed in the same tank. *N* = 17 2.2–7 and 27 NTG at 8 months. (**d**) After being removed from the swim tunnel, 9 month old fish did not show any significant difference in proportion of transitions into slow (left), intermediate (middle) or fast (right) movements. *N* = 12 fish per genotype. (**e**) After being removed from the swim tunnel, 12 month old 2.2–7 zebrafish showed a significant increase in the proportion of transitions into slow movements and a corresponding significant decrease in the proportion of transitions into fast movement. There was no change in transition into intermediate movement. N = 13 fish per genotype. All data are shown as mean +/− standard deviation; *P < 0.05, **P < 0.01, ***P < 0.001 and ****P < 0.0001

To assess the neuro-muscular integrity of the 2.2–7 transgenic zebrafish, swimming endurance was tested using a swim tunnel, the aquatic equivalent to a treadmill [31]. At 9 months of age more 2.2–7 transgenic zebrafish failed to maintain swimming at the maximum flow rate as compared with their NTG clutchmates (Fig. 6a). Despite decreased body mass during early development, body mass and body size were not significantly different between adult transgenic and NTG groups from 9 months of age (Additional file 2: Figure S1). Spontaneous swimming was observed immediately following swim tunnel testing, but no difference was observed between the two groups (Fig. 6d). The swim tunnel test was repeated with the same cohort of zebrafish at 12 months of age, and the ability to swim at maximum speed continued to decrease in the 2.2–7 zebrafish (Fig. 6b). Interestingly, at 12 months 2.2–7 zebrafish now showed defects in spontaneous swimming behaviour following the swim tunnel testing. 12-month-old zebrafish showed an increase in the proportion of times transitioned into slow speed movements and a concomitant decrease in the proportion of times transitioned into fast speed movement, as compared to NTG clutchmates at the first-time point following swim tunnel testing (Fig. 6e).

Adult survival was also monitored from 8 months post-fertilisation onwards. By 17 months post-fertilisation, survival rates of the 2.2–7 transgenic zebrafish were significantly reduced in comparison to their NTG clutchmates (44% vs 100% survival respectively; Fig. 6c). A zebrafish was defined as having reached end-stage once it had lost the ability to maintain normal swimming (showing signs of paralysis) to the extent where it was no longer able to obtain food. End-stage 2.2–7 zebrafish displayed severe wasting in the body muscle region and had very poor locomotor skills (Additional file 3: video 1, no NTG zebrafish displayed this wasting phenotype.


Additional file 3: End-stage 2.2–7 zebrafish video. (MP4 15919 kb)


### Muscle atrophy and motor neuron loss in *C9orf72* zebrafish

**Fig. 7:**
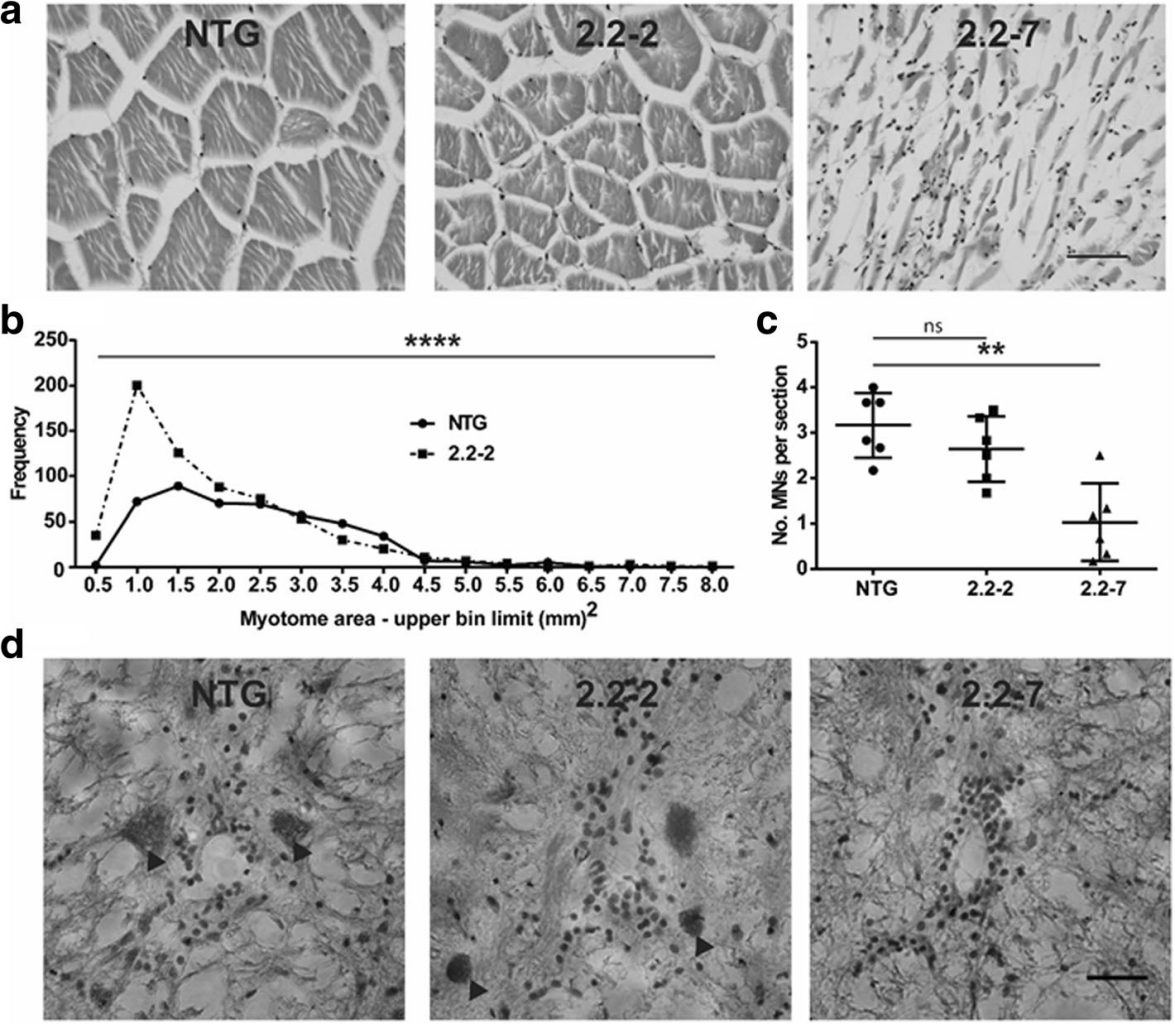
*C9orf72* model zebrafish display muscle atrophy and motor neuron loss. (**a**) Representative H&E staining of zebrafish epaxial muscle (body muscle) myotomes. Scale bar = 50 μm. (**b**) Frequency distribution of 2.2–2 and NTG myotome sizes. N = 6 individual zebrafish per genotype. (**c**) Motor neuron counts show that 2.2–7 zebrafish have significant motor neuron loss compared to NTG. N = 6 individual fish per genotype. (**d**) Representative H&E staining of zebrafish spinal cord sections, motor neurons are denoted by arrowheads. Scale bar = 25 μm. Myotome size data are shown as the frequency of myotome sizes binned into defined ranges, motor neuron count data are mean +/− standard deviation; *P < 0.05, **P < 0.01, ***P < 0.001 and ****P < 0.0001

Progressive muscle atrophy is observed in all ALS patients. Similarly, end-stage 2.2–7 zebrafish muscle displayed widespread severe atrophy, muscle fibres were disorganised, and a large increase in nuclei was observed (Fig. 7a). The muscle of 2.2–2 zebrafish displayed more subtle changes, with myotomes being significantly smaller and more numerous as compared to NTG muscle (Fig. 7a+b). We did not quantify end-stage 2.2–7 zebrafish muscle fibre size, as their myofibres were too disorganised to discern individual myotomes.

In ALS patients, the underlying molecular pathology ultimately leads to motor neuron death. Similarly, significant loss of ventral horn motor neurons was observed in end-stage 2.2–7 zebrafish as compared with NTG controls (Fig. 7c+d). A small, non-significant reduction in motor neurons was observed in 2.2–2 zebrafish as compared with NTG controls.

### Heat shock stress response is activated by *C9orf72* expansions

**Fig. 8:**
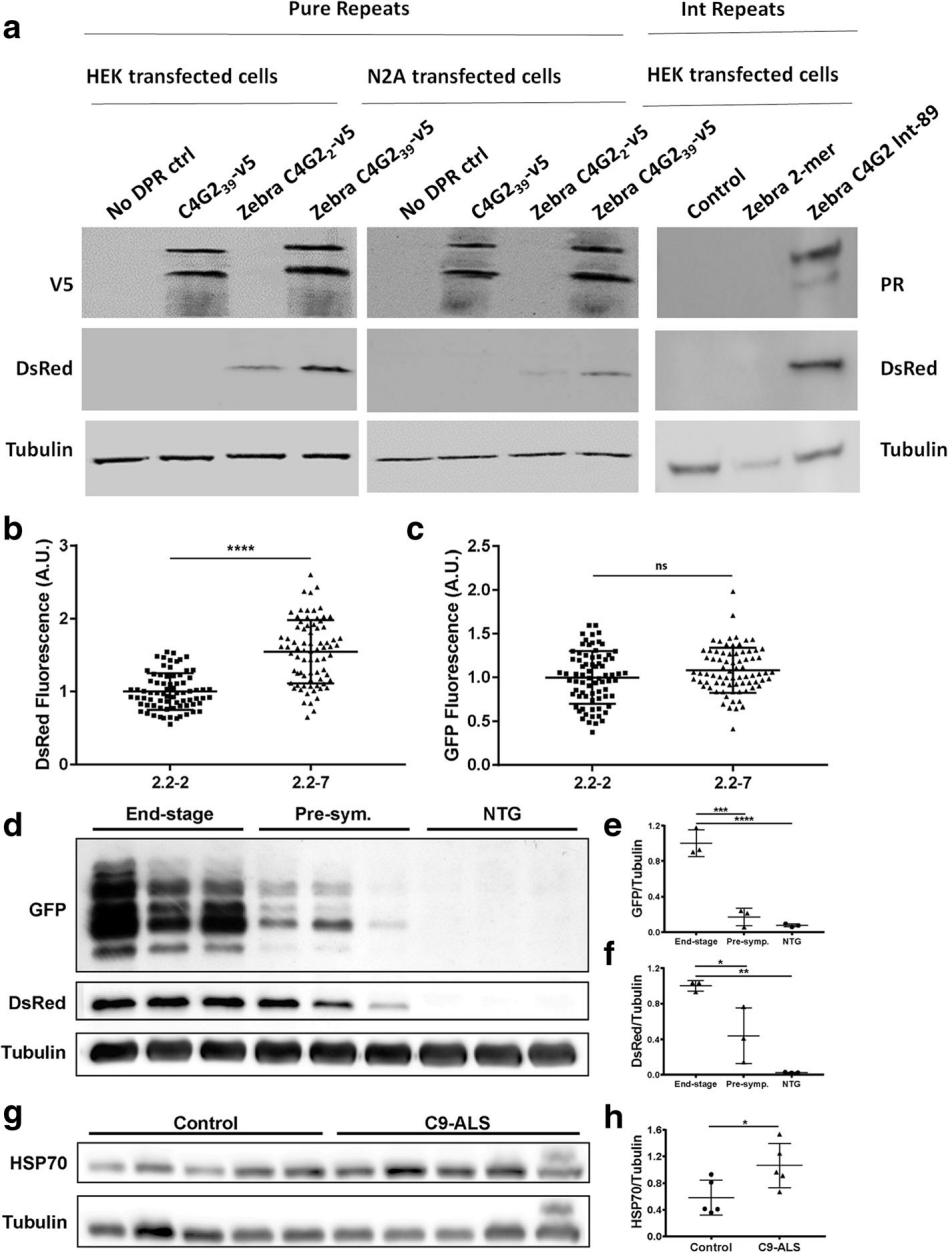
Heat shock stress response activation is induced by *C9orf72* expansions. (**a**) In lysates from both HEK 293 T and N2A cell lines, DsRed levels are higher in cells transfected with 39 C4G2 pure repeats (Left and middle panel) or 89 C4G2 interrupted repeats (Right panel) compared with those transfected with only 2 C4G2 repeats. (**b**) At 5dpf, 2.2–7 zebrafish show significantly higher DsRed fluorescence than 2.2–2 zebrafish. *N* = 75 2.2–2 and 76 2.2–7 individual zebrafish. (**c**) At 5dpf, GFP fluorescence is not significantly different between 2.2–7 and 2.2–2 zebrafish. N = 75 2.2–2 and 76 2.2–7 individual zebrafish. (**d**) In end-stage 2.2–7 zebrafish brains, levels of GFP tagged DPR and DsRed proteins are increased compared with pre-symptomatic 2.2–7 and NTG. (**e**) Quantification of GFP tagged DPR protein normalised to tubulin in adult zebrafish brains. N = 3 adult brains per condition. (**f**) Quantification of DsRed protein normalised to tubulin in adult zebrafish brains. N = 3 adult brains per condition. (**g**) In human cerebellum samples, HSP70 protein levels are higher in C9-ALS patients as compared to non-neurological-disease controls. *N* = 5 samples per group. (**h**) Quantification of HSP70 protein levels normalised to tubulin in human cerebellum. All data are shown as mean +/− standard deviation; *P < 0.05, **P < 0.01, ***P < 0.001 and ****P < 0.0001

Heat shock proteins are upregulated in response to the presence of aberrant cellular proteins [4, 10]. We hypothesised that the low-complexity structure of DPR proteins might drive activation of the heat shock response (HSR). To test this, we transfected both HEK 293 T and N2A cells with *C9orf72* expansion containing pure HRE and interrupted HRE constructs. The repeats were expressed in tandem with a *hsp70* promotor driving a DsRed gene, as a readout of heat shock response activation. As DsRed is more stable than hsp70, it allows more sensitive detection of small but chronic HSR activation [24]. In both HEK and N2A cells, cells transfected with 39 C_4_G_2_ pure repeats (left two panels) or 89 interrupted repeats (right sided panel) showed strong RAN-translated V5-tagged DPR or ATG driven PR-tagged DPR production and markedly higher DsRed production (Fig. 8a). In contrast, cells transfected with only 2 C_4_G_2_ repeats displayed no RAN-translated DPRs and less or undectable DsRed production. As expected cells transfected with 39 C_4_G_2_ repeats but no *hsp70:DsRed* heat shock readout, produced abundant RAN-translated DPRs but no DsRed protein.

To assess differences in HSR activation in the more phenotypically severe 2.2–7 zebrafish vs the less severe 2.2–2 zebrafish, we screened 5 dpf zebrafish for DsRed (produced via *hsp70* promotor activation). The more severe 2.2–7 zebrafish showed significantly increased DsRed fluorescence in comparison to 2.2–2 zebrafish at 5dpf (Fig. 8b). Importantly, GFP fluorescence (from GFP-tagged DPRs) was not significantly different between 2.2–7 and 2.2–2 zebrafish (Fig. 8c).

To assess how HSR activation changes as phenotypic severity increases, we examined GFP and DsRed production in adult zebrafish brains, from 3 end-stage 2.2–7 zebrafish (ages 15, 15 and 19 months), 3 pre-symptomatic 2.2–7 zebrafish (all aged 7 months) and 3 NTG zebrafish (age matched to end-stage). Pre-symptomatic was defined as fish which did not show any overt swimming or muscle abnormalities. GFP tagged DPRs were increased in the brains of end-stage zebrafish in comparison to the brains of pre-symptomatic zebrafish (Fig. 8d+e). Similarly, DsRed also increased in the brains of end-stage zebrafish in comparison to the brains of pre-symptomatic zebrafish (Fig. 8d+f), thus suggesting an association between DPR production and HSR induction.

Finally, we examined whether HSR activation could occur in the presence of the DPR proteins in cerebellar post-mortem tissue from *C9orf72* ALS patients. Cerebellum tissue was selected to study the effect of DPRs on HSR, as previous reports indicate cerebellum tissue consistently shows a high DPR load [2, 9, 21, 22]. Firstly, we confirmed that DPR species are expressed in the cerebellum of these C9-ALS patients (Additional file 4: Figure S2. Next, HSP70 protein levels in human cerebellum were assessed using western blotting. C9-ALS patients had significantly higher cerebellar levels of HSP70 as compared with non-neurological-disease controls (Fig. 8g+h). Taken together, our data demonstrate that *C9orf72* expansions activate the heat shock response.

**Fig. 9:**
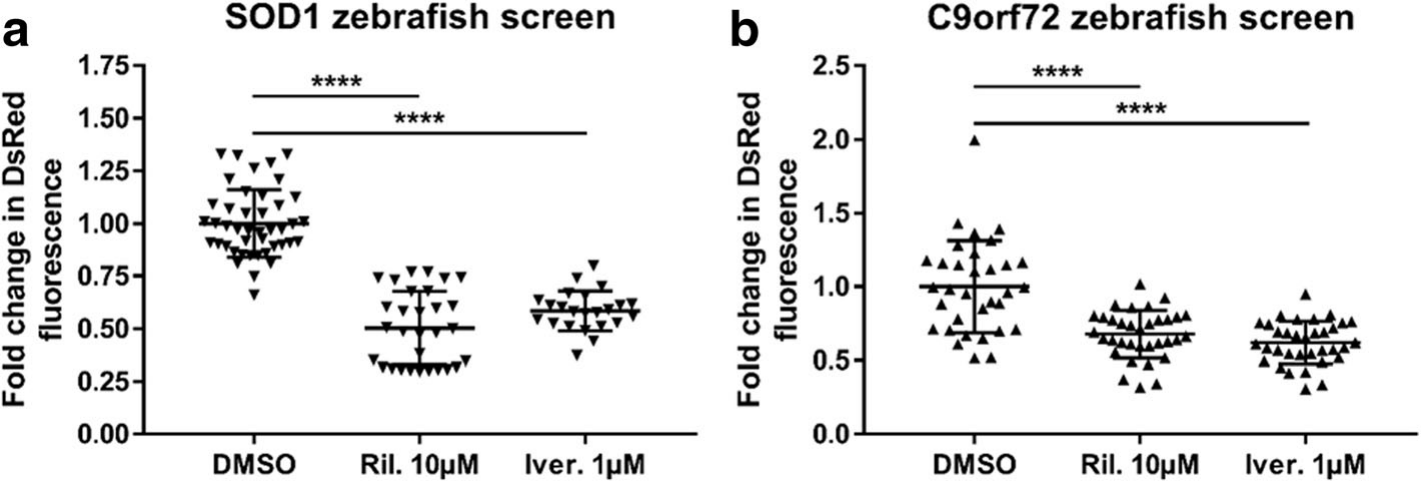
Riluzole and Ivermectin modulate HSR in sod1 and C9orf72 zebrafish. (**a**) Treatment with either 10 μM riluzole or 1 μM ivermectin from 2 to 5 dpf resulted in a significant reduction in DsRed fluorescence in *sod1* zebrafish, as compared to DMSO treatment. *N* = 30 riluzole treated, 23 ivermectin treated and 43 DMSO treated individual zebrafish. (**b**) Treatment with either 10 μM riluzole or 1 μM ivermectin from 2 to 5 dpf resulted in a significant reduction in DsRed fluorescence in *C9orf72* zebrafish (2.2–7 line), as compared to DMSO treatment. *N* = 34 riluzole treated, 34 ivermectin treated and 33 DMSO treated individual zebrafish. All data are shown as mean +/− standard deviation; *P < 0.05, **P < 0.01, ***P < 0.001 and ****P < 0.0001

Both *C9orf72* and *SOD1* ALS zebrafish models express a *hsp70* promotor which drives DsRed protein production. Cell stress from a variety of insults increases the drive on the *hsp70* promotor, and upregulation of the HSP70 protein has been reported in neurodegenerative disorders such as multiple sclerosis and, in the present study, ALS [19, 23, 27]. Therefore, in our ALS zebrafish models, the abundance of DsRed produced via hsp70 promotor activation is used as a readout of cellular stress. Drugs which reduce cellular stress, and thereby reduce *hsp70* promotor mediated DsRed production can be identified by treating zebrafish with the drug from 2 to 5 dpf, and then measuring DsRed levels in a fluorescence plate reader [25]. To date, thousands of compounds have been tested using this drug screening paradigm in *SOD1*-ALS zebrafish models (current authors, data not shown). Ivermectin is a compound which was identified as one of the most efficacious drugs in the *SOD1* zebrafish screen. In *SOD1* zebrafish ivermectin treatment reduced the level of HSR activation (as measured by DsRed fluorescence) to a similar degree as riluzole (the only disease modifying treatment currently prescribed for ALS; Fig. 9a). Thus, in *C9orf72* zebrafish ivermectin treatment also resulted in a significant reduction of HSR activation, and compared with the *SOD1* zebrafish screen, the efficacy of ivermectin was comparable to that of riluzole (Fig. 9b). Therefore, these data suggest that cross over between *SOD1* and *C9orf72* pathology may allow for a single treatment to be efficacious in both disease forms.

## Discussion

We have generated *C9orf72*-related ALS model zebrafish which stably express interrupted C_4_G_2_ expansions and exhibit RNA foci and DPR pathology. These zebrafish accurately recapitulate key aspects of the behavioural, cognitive, motor defects and reduced survival associated with C9-ALS/FTD. Additionally, these zebrafish have been utilised to identify that poly(PR) DPRs form higher molecular weight species. Furthermore, these *C9orf72* zebrafish were used in conjunction with human cell lines and human post-mortem tissue to identify that *C9orf72* expansions activate the HSR. Finally, we identified that ivermectin treatment reduces cell stress HSR activation in both *SOD1* and *C9orf72* zebrafish models. The novel aspects of the *C9orf72* zebrafish model we have generated here are compared and contrasted to other *C9orf72* in vivo models in Table 1.

**Table 1:** Comparison of Stable *C9orf72* stable mouse and zebrafish mutants showing the distinct phenotype and utility of each model

DNA Construct	Species	RNA Foci	Sense RAN DPR	Antisense RAN DPR	ALS phenotype	FTD like phenotype	Stable or Transient	Phenotype amenable to drug screen	Efficacy of riluzole	Reference
BAC HRE	Mouse	Yes	Yes	No	Yes	Yes	Stable	No	Not tested	[14]
BAC HRE	Mouse	Yes	Yes	No	Yes	Yes	Stable	No	Not tested	[20]
BAC HRE	Mouse	Yes	Yes	No	No	No	Stable	No	Not tested	[29]
BAC HRE	Mouse	Yes	Yes	No	No	No	Stable	No	Not tested	[30]
C9 K/O	Mouse	NA	NA	NA	No	No	Stable	No	NA	[1, 13, 17, 35]
C9 K/D	Zebrafish	NA	NA	NA	Axonal growth defect	No	Transient	No	Not tested	[6]
C9 K/O	Zebrafish	NA	NA	NA	No	No	Stable	No	NA	[34]; (Schmid, Hruscha, Haass, unpublished)
C9 HRE	Zebrafish	Yes	No	No	No (Mild cardiac phenotype)	No	Stable	No	Not tested	[28]
C9-ATG GA	Zebrafish	Yes	No	No	No (Severe cardiac phenotype)	No	Stable	No (lethal)	Not tested	[28]
C9 -ATG HRE	Zebrafish	Yes	Yes	Yes	Yes	Yes	Stable	Yes	Yes	Current manuscript

*NA* Not applicable, *K/O* Knockout, *K/D* Knockdown, Grey boxes represents the features that represent similarity to human ALS/FTD or utility in the high throughput screening of novel therapeutics

The zebrafish model presented here lends support to a gain of function as the toxic mechanism underlying *C9orf72* ALS/FTD. Our data are consistent with several other studies in animal models showing toxicity mediated by RNA foci and DPRs [5, 20, 26, 38], including two independently generated *C9orf72* zebrafish models [18, 28]. Furthermore, our data are consistent with four independently generated *C9orf72* knock-out mice and one knockout zebrafish model, none of which display any motor or neurodegenerative changes, arguing against haploinsufficiency as a major contributor to *C9orf72* ALS/FTD [1, 13, 17, 35], (Schmid, Hruscha, Haass, unpublished). In contrast, decreased *C9orf72* transcript levels have been reported in the CNS of G_4_C_2_ expansion bearing patients, and morpholino mediated knockdown of *C9orf72* transcripts have been linked with motor deficits in zebrafish [6, 11]. However, morpholinos notoriously have off-target effects and may fail to mimic the phenotypes observed in stable knockout mutant zebrafish [16]. Thus, the current body of evidence is heavily weighted towards RNA foci/DPR mediated gain of function toxicity in *C9orf72* expansion pathobiology.

Western blotting of zebrafish lysates revealed that multiple lengths of GFP-tagged DPRs are produced (including the predicted 48KDa full length peptide) producing a laddered appearance. Both sense and antisense DPR were detected and were produced by both conventional and RAN-translation. Detection of species of varying MW has also been reported during RAN-translation of CAG repeats [42], and during RAN-translation of GGGGCC in C9-ALS patients [43]. More RAN-translation mediated bands were detected in 2.2–7 zebrafish compared to 2.2–2. Interestingly, poly(PR) species were detected at higher MWs than other DPR species, and it will be important to investigate whether the tendency of poly(PR)s to form high MW species is related to the potent in vivo toxicity. This suggests that RAN-translation blocking agents aimed specifically at inhibiting HMW poly(PR) formation may be an important therapeutic avenue to pursue.

The more severe 2.2–7 zebrafish line showed embryonic onset motor defects and evidence of cognitive abnormalities, thus suggesting that DPR/RNA foci pathology is adversely affecting not only the motor unit, but also cognitive function; consistent with the spectrum of ALS/FTD in *C9orf72* patients. Assessment of centre avoidance behaviour indicated that 2.2–7 zebrafish showed an unwillingness to explore, similar to *C9orf72* mice assayed with the open field paradigm [20]. Early mortality is also observed in the more severe 2.2–7 zebrafish, indicating that motor and cognitive defects detectable at the embryonic stage later become severe enough to impact upon survival. Reduction in body weight was observed in 2.2–7 zebrafish at the larval stage, however this later recovered by adulthood, suggesting that the reduction was due to retardation of the growth process rather than tissue degeneration. Indeed, it is possible that slowed growth during early development of the 2.2–7 line may be due to the observed motor defects reducing access to food.

Swim tunnel performance of the 2.2–7 zebrafish was significantly poorer than that of their NTG clutchmates at both 9 and 12 months. Swim tunnel performance is mainly indicative of the neuromuscular integrity of zebrafish body muscle, however cardiovascular involvement cannot be ruled out. Small differences in spontaneous swimming behaviour observed at 9 months became significantly different at 12 months, indicating progression of phenotypic severity. Disease progression was also confirmed when the same 2.2–7 swim tunnel tested zebrafish displayed clear signs of muscular atrophy and became unable to swim, necessitating culling. None of the NTG clutchmates showed this progressive atrophic phenotype. By 17 months of age over 50% of the 2.2–7 zebrafish required to be culled, however most of the remaining zebrafish appeared healthy. This indicates a heterogeneity in progression of phenotype in the 2.2–7 zebrafish, and suggests that genetic, epigenetic or other factors may modulate the disease phenotype. Indeed, this phenomenon may explain why the 2.2–2 zebrafish model present a less severe phenotype. Similar variability in phenotypic severity has previously been reported in BAC mice expressing the *C9orf72* gene [20].

Abnormal muscle histology was observed in both 2.2–2 and 2.2–7 zebrafish. Generally muscle fibres were smaller and more numerous in the transgenic zebrafish, consistent with atrophy and attempted regeneration. Significant motor neuron loss was also observed in 2.2–7 zebrafish and a trend in the same direction was observed in the 2.2–2 zebrafish. At this point it is not possible to determine whether the degeneration of the neuromuscular unit was neurogenic or myogenic in origin, and given that it is now known that DPR may transmit from cell to cell there may well be a contribution to toxicity from both tissues [41].

Previous transient RNA-injection zebrafish models suggest that G_4_C_2_ RNA is sufficient to cause activation of apoptosis and motor axonopathy [18, 36]. It is important to note that transient RNA-injection models express RNA in much higher concentrations than would be observed in stable animal models, therefore the observed pathology is less likely to be reflective of pathology under physiological conditions. The RNA-injection zebrafish were not characterised longitudinally as the transgene is only expressed transiently (typically for 1–3 days). Additionally, an independently generated stable zebrafish model has previously shown that 80 X (G_4_C_2_) RNA or poly(GA) DPR expression leads to pericardial oedema related toxicity at 4 dpf, but no neurological or motor phenotype was reported at any time point [28]. In contrast, over a comparable time period (5 dpf), the zebrafish presented here showed both motor and cognitive dysfunction. Additionally, our zebrafish model survived to adulthood and displayed adult-onset motor defects which eventually lead to motor neuron loss and death, thus recapitulating key features of human ALS/FTD over multiple time points. If model organisms are to be reliable in terms of the mechanistic insights or the therapeutic targets they generate, then they must reflect disease features accurately. Future models should include as many disease relevant features as possible until the exact mechanisms of *C9orf72* expansion toxicity are better understood.

HSP70 protein levels were found to be increased in C9-ALS patient cerebellar tissue. Consistent with previous reports, these cerebellum samples were found to have a substantial DPR load, thus DPRs may mediate cerebellar HSR activation [2, 9, 21, 22]. Activation of the HSR as measured by DsRed protein expression under the control of the *hsp70* promotor, was found to be higher in cells transfected with 39 C_4_G_2_ repeats compared to cells transfected with only 2 C_4_G_2_ repeats, thus indicating that *C9orf72* expansions of a pathological length are required for activation of the *hsp70* promotor. Additionally, activation of the HSR as measured by DsRed protein expression, was higher in 2.2–7 zebrafish compared with 2.2–2 zebrafish. However, in the same fish GFP fluorescence was not significantly different, indicating that the total amount of DPR in each of the 2.2-zebrafish lines is equivalent. The reason for a greater activation of HSR in 2.2–7 could be due to the differential pattern of DPR expression between the two zebrafish lines. Variability in transgene copy number is unlikely to underlie the difference in DsRed production between the 2.2–7 and 2.2–2 zebrafish, as GFP levels between the two are not significantly different. DsRed and GFP tagged DPRs also progressively increased in the brains of end-stage zebrafish, indicating that DsRed production positively correlates with both DPR production and disease severity.

Furthermore, *C9orf72* and *SOD1* ALS zebrafish models were both validated as good quality drug screening models by demonstrating reduced cell stress HSR activation following treatment with riluzole. More importantly, *SOD1* zebrafish identified the compound ivermectin as reducing cell stress HSR activation, and this finding was then mirrored in *C9orf72* zebrafish, further suggesting that there is cross over between *SOD1* and *C9orf72* pathology.

## Conclusion

The stable transgenic *C9orf72* zebrafish model we have generated exhibits RAN-translation of DPRs, motor neuron loss, muscle atrophy, motor impairment, cognitive abnormalities and reduced adult survival. Thus, our zebrafish model accurately recapitulates the more complex aspects of human C9-ALS/FTD pathobiology, which is essential for studying the underlying mechanisms of ALS/FTD. In addition to all previous in vivo models of any species, our zebrafish model offers the unique benefit of being validated for screening of therapeutic compounds. Using this *C9orf72* zebrafish model we have identified novel insights into the pathogenesis of C9-ALS/FTD. Specifically, we identified that poly(PR) DPRs are RAN-translated into higher molecular weight species compared to other DPRs, which may explain the greater in vivo toxicity of this DPR species. Blocking formation of HMW poly(PR) proteins may therefore represent a novel therapeutic avenue. Additionally, we identified that the heat shock response is activated by *C9orf72* expansions, indicating that protein chaperone machinery may modify the disease course through a role in attempted preservation of protein homeostasis. Finally, by tandem drug screening with *sod1* and *C9orf72* zebrafish we identified that ivermectin may hold therapeutic potential in both of these forms of ALS. Rapid drug screening and validation of hits in zebrafish models of multiple ALS disease genes will be a powerful drug-discovery tool going forward.

## Additional files

Additional file 1: Sequence of transgene injected to create C9-HRE transgenic zebrafish. (PDF 120 kb)

Additional file 2: **Figure S1.** Body mass and body length were not significantly different at the time of swim tunnel testing. (**a**) At 9 months old, body mass of the 2.2–7 and NTG zebrafish tested in the swim tunnel was not significantly different. *N* = 12 zebrafish per genotype. (**b**) At 9 months old, body length of the 2.2–7 and NTG zebrafish tested in the swim tunnel was not significantly different. N = 12 zebrafish per genotype. (**c**) At 12 months old, body mass of the 2.2–7 and NTG zebrafish tested in the swim tunnel was not significantly different. *N* = 13 zebrafish per genotype. (**d**) At 12 months old, body length of the 2.2–7 and NTG zebrafish tested in the swim tunnel was not significantly different. N = 13 zebrafish per genotype. All measurements were carried out ~ 40 min after removal from the swim tunnel (5 min rest, 30 min spontaneous behaviour recording and another 5 min of rest). (TIF 360 kb)

Additional file 4: **Figure S2.** Poly(GA) and poly(GP) DPR proteins are produced in cerebellum of C9orf72 patients. (**a**) Dot blots of grey matter cerebellum samples from *n* = 5 control, sALS and C9orf72 patients each. Immunoblotting with an antibody against tubulin reveals mostly even loading amongst the numerous samples. Immunoblotting with an antibody against poly(GA) reveals that C9orf72 patients express abundant poly(GA) DPRs, whereas control and sALS samples do not. And immunoblotting with an antibody against poly(GP) reveals that C9orf72 patients express abundant poly(GP) DPRs, whereas control and sALS samples do not. (**b**) Quantification showing that in cerebellum grey matter, significantly higher poly(GA) signal is detected in C9-ALS samples in comparison to control samples, when normalised to tubulin. (**c**) Quantification showing that in cerebellum grey matter, significantly higher poly(GP) signal is detected in C9-ALS samples in comparison to control samples, when normalised to tubulin. Con: Control, sALS: sporadic-ALS, C9: C9orf72-ALS. (TIF 485 kb)
