# Supplementary material for: Stable transgenic C9orf72 zebrafish model key aspects of the ALS/FTD phenotype and reveal novel pathological features
Source: Acta Neuropathol Commun. 2018 Nov 19;6:125. doi: 10.1186/s40478-018-0629-7 (PMC6240957; doi:10.1186/s40478-018-0629-7)
Supplement: Supplementary file 2 — Sequence of transgene injected to create C9-HRE transgenic zebrafish. (PDF 120 kb) [file 40478_2018_629_MOESM1_ESM.pdf]

ALS/FTD associated C9orf72 expansions activate the heat shock response and induce SOD1 proteinopathy.

Acta Neuropathologica

Matthew P. Shaw, Adrian Higginbottom, Alexander McGown, Lydia M. Castelli, Evlyn James, Guillaume M. Hautbergue, Pamela J. Shaw, and Tennore Ramesh

Sheffield Institute for Translational Neuroscience, University of Sheffield, 385 Glossop Road, Sheffield, S10 2HQ, United Kingdom.

The Bateson Centre, Firth Court, The University of Sheffield, Western Bank, Sheffield, S10 2TN, United Kingdom

t.ramesh@sheffield.ac.uk

Online resource 1:

E Supplementary Doc-1

- ISceI meganuclease restriction site
- Zebrafish ubiquitin promotor
- GFP
- (C4G2)89 C9orf72 expansions
- Heat shock promotor 70
- DsRed

NB: Due to difficulties in sequencing GC rich DNA we were not able to obtain a sequencing read which spanned the entire C4G2 expansion, therefore expansion size is listed as 89 C4G2 repeats which is the minimum known size.

Listed below is the entire DNA sequence contained in a single transgene copy -

CAGGGTAATAGCTTTTAATCTCAAAAAACATTAAATGAAATGCATACAAGGTTTT
ATCCTGCTTTAGAACTGTTTGTATTTAATTATCAAACTATAAGACAGACAATCTA
ATGCCAGTACACGCTACTCAAAGTTGTAAAACCTCAGATTTAACTTCAGTAGAAG
CTGATTCTCAAAATTGTTAGTGTCAAGCCTAGCTCTTTTGGGGCTGAAAAGCAAT

CCTGCAGTGCTGAAAAGCCTCTCACAGGCAGCCGATGCGGGAAGAGGTGTATTA
GTCTTGATAGAGAGGCTGCAAATAGCAGGAAACGTGAGCAGAGACTCCCTGGTG
TCTGAAACACAGGCCAGATGGGTTTAAACGAATTCGTCGAGACCAGCAAAGTTC
TAGAATTTGTCGAAACATTTATGTTATATATTTCCTGAAAAAAATTCTGAGTAAG
TTCTTAAGTGTTATTGCCAGCAACATAAACAACAGACGGCAAAATGAATAAATG
ATAACAAAGCAGTAGGCTTAAATAAACCTAATTTTTATAGGCTGTTCTCTACAAC
CCTCAAACAGTGATTAGTTTTGTACTTATAAACTTGCCCTTTCATTCATATTTCAA
GAAAATTGGTTCAGAAGATCTGGATATTCTAGCAGTTGTTCAAGCTCATGGAGGG
ATCAGTGACCTGATTCCACAATGACTAGGCCTAATCCAGAAATTAGATGACTGTC
AACATAAAAAGGCACAGCACTCACTAGCTGCCCTATATATTTTATTATATTTTAC
ATATATTATTTTATTTATTTAGCTCTGAGTGCTGTACTTTCTGGTTAAAGAAAACT
GCTTACAACAGCTAACCTGTACTACCTCAGGCTCAGGGAATTTGGAACAGGTTTG
TCTGGTTTGTTTCTTTAACCATGCATGCTTGTTTTCAACTATGGCAACACAGTCAC
ATGGGACATTACAGAAATGATTTGTCGATGACATGCGACTTTTCTTTAATAAAGC
GCAAAGATCCCAAAAAGCAAACTTTTAACAAAAATCATATAATTATATTTTCAAT
CCAGCTTTGTAGCAACTTTGTGCTGCTGTTCACTCAGCAACAGATAGTCAGTATA
AGGTCAGTGTGTCTCAAAGCAGTGCCATCTGTTTCACACATTGCGTTCTATATAT
AAGTGTGCTGGTTGACACGACACTGTATAAGGCCTAGGCTAAAACACAAACAAT
GTAGAATGACACTGTGTTTTTTTTGTAAACAAATGTTGTTTTTGGTTAAACATCTT
TGTGAAAACATCCTCCTGTCATGTATTTGCTATATTCAAATGTTAAACCCGTGCA
GAATAGAACATATACAAAAAAAAACAACACAACACATTTTTAAACATTATTAAA
TATCAAGTATTGCTGGCAGTTCTGTTTCTGTTTTACAGTACCCTTTGCCACAGTTC
TCCGCTTTTCCTGGTCCAGATTCCACAAGTCTGATTCACCAATAGCAAAGCGAAT
AAACAACCAAAGCAGCCAATCACTGCTTGTAGACTGTCCTGCGAGACCGGCCCA
TTCCAGCACATTCTGGAAACTTCCTTTATATGATAATTATAAATACATTTAAATTA
TTGATACAAAACATGTAATTCCTAGAACATAACCATAGCAATCATTAGTTTTCAG
GGTAATTATGTATTTTTAGGATTTGACTGCGGAAAGATCTGGTCATGTGACGTCT
CATGAACGTCACGGCCCTGGGTTTCTATAAATACAGTAGGACTCTCGACCATCGG
CAGATTTTTCGAAGAAGAAGATCAGTTTCAGGAGCCGTACTGTTCCGTTTCAACG
CAAATATTAACGGTAAGAGCGAATTTCCTAGTTTGTTTTCATGCCATTCTTTAAAA
CCATAGCGTATTACTTTAATTATAGTAAACTTTCGCTTTCTTTATTACAAGAGACG
TTTTGTGTTGATTCTCCGCGGACATTTTCGGTCAGACAATCAGAAAATGACCGCG
GAGGACCAGTAACTTGCATTACACGTAAGTTAAATCTTCGTGTATTAAAATGGTT
AGGTTGTTAACGTCAAATAGGTTACCGTGTTTGCGTGTGATCAGGTTGGTTTTGTT
AGATTTTTGTCAGTATTTTTAATTTATTTGTTTTAGTTTATTTATTTTTTTTGCTGA
ATCATAGTTTGTGAACAAAGAACCCGGATGTTACATACAGTACAGCCGCCATGTT
ACAGAGAGTTATAACTTAATCATTTTAAAAATAATTTTGCCTTACTTTTAGTTTGT
CATGTTGAGAAATGAGGAAATGTTAAAATGAGGAAATATCCAATTAATTTAATA
TATCAAAATAATCCATGATTACAATGCACTGAACTGGAGAAAATTAAGATGTTTT
CTAGTGTCATGAAACAAATGTAAGAGATGTACATTGTAGATGTTTTATGTCAAGA
ATTGGCTAGTTGATGCAGCATACTGGCGATACTCAGTTGTAATAACAGTAACGTT
ACATGTTAATAGACTACTGAGTATGCTGTTCTGTCTATGTATGCTCTGTAAGCTAC
GAGAAGGACTTTTTTAAACAGTAAAGGGTGCAATATTTTTACAAATTGAATTAAA
TAAAGGCTGTCTATTAAGTAATATGCTTGATATTTTTCTTACTTGATCGGAAATAA
GAAAAAATATAAACGTTGTTGCTCTAAAAATCCTAGTTCAGTTTAGCCAACCACA
AATACCTTTTTGTTCCTCCAACAGTTTTTTTTTCTTCTCTATAATATTTGGCAGTCT

ATAGTACTCCAAATGTTTCCCCACAGTCTAACTAATTGGTACAGCCAAAATCATG
ACACTTATTGCAATAATAATTTTGGTTCATTGGCATTGTTGATAGCCTGTGCCACT
AATATGGTCGATTGATCATGCTTCAGGAAGAAAACTATATTGTTTGATGTAAGAT
TATTAAATCTTCACCTGCTTCCATTACAAACTATTCCCATCTTATTGAATTCTGGT
ATGTCTTAAAGGATTAGTTCACTTCCCAAATCAAAATTTACTTAGCCTTTTTTCAT
CCATGATCCCTTTTTTTCATCATTAATGAAGAAATTGTTTTTGAAAAAGTTTCAAG
ATTTTTTTCTCTATATTGTGGAGCTTGTTAGTTTAAAATTCCAAAATGCAATATGT
GGCTTCAAATGGTTCTAAATGATCCCAGTCAAGGAATAACAGTCTTATCTAATGA
AACCATTAGACCTTTAAAAATAAAAAAAATAAAAGTATTTATTTTTAAATGACTG
AGTGATTAAGTTGAATTTCAGCGTTTCCTTACTGTGTAGAAGTCCTTCCTTACTGG
CCCCACCCTTTGGTTCTCTGCCAATCTGCTACCTAATGTAATGTTGTGGAACATTA
TTATTCTTTATTTCTTAATTTTTTATTTTTTATTTTAAAACAATGTAAACTGCACAG
ATGTGCAGTTTGTTTAAAATGGCCAATGCTTTGGAAATGCATGACATAATTAGAT
TTCATGATGCACAAAGCCAAATCTCAGAGCTTGTGCAAAATGAGCTATCATTTCA
CTAGGTAAGACCCTAAATTTTCATATAGGATCATTTGGACAATTTTGCTGCAGGT
AAAATGCATTCTATAGTCCACTGTCAGCCATTGTTTTGGATAGTATTTATTTTTCT
CTACAAGTATAGTCAATAGTTTTCTATTATTTTAAAGGTTTGTAACATTTAAGGGT
GACCAAATGCAAAGTAAAATTTCATTTTCGGGTGAACTATCTCGTTTAACATGGG
AGAAGTGCAAAACATACATTATTGGCTAGAACATTGTAGTATTTTTTAAATGGAA
ATGTGTGATTGCTAATCTTACTTTGAATTTGTTTACAGGGATCTATAACTTCGTAT
AGTATACCTTATACGAAGTTATCGAGCTCATAACTTCGTATAGGATACTTTATAC
GAAGTTATAACGTCGAGATAACTTCGTATAGCATACATTATACGAAGTTATAGGA
TCAATGGTGAGCAAGGGCGAGGAGCTGTTCACCGGGGTGGTGCCCATCCTGGTC
GAGCTGGACGGCGACGTAAACGGCCACAAGTTCAGCGTGTCCGGCGAGGGCGAG
GGCGATGCCACCTACGGCAAGCTGACCCTGAAGTTCATCTGCACCACCGGCAAG
CTGCCCGTGCCCTGGCCCACCCTCGTGACCACCCTGACCTACGGCGTGCAGTGCT
TCAGCCGCTACCCCGACCACATGAAGCAGCACGACTTCTTCAAGTCCGCCATGCC
CGAAGGCTACGTCCAGGAGCGCACCATCTTCTTCAAGGACGACGGCAACTACAA
GACCCGCGCCGAGGTGAAGTTCGAGGGCGACACCCTGGTGAACCGCATCGAGCT
GAAGGGCATCGACTTCAAGGAGGACGGCAACATCCTGGGGCACAAGCTGGAGTA
CAACTACAACAGCCACAACGTCTATATCATGGCCGACAAGCAGAAGAACGGCAT
CAAGGTGAACTTCAAGATCCGCCACAACATCGAGGACGGCAGCGTGCAGCTCGC
CGACCACTACCAGCAGAACACCCCCATCGGCGACGGCCCCGTGCTGCTGCCCGA
CAACCACTACCTGAGCACCCAGTCCGCCCTGAGCAAAGACCCCAACGAGAAGCG
CGATCACATGGTCCTGCTGGAGTTCGTGACCGCCGCCGGGATCACTCTCGGCATG
GACGAGCTGTACAAGAAAGCTGGGTCTAGATATCTCGAGG
CCCCGGCCCCGGCCCCGGCCCCGGCCCCGGCCCCGGCCCCGGCCCCGGCCCCGG
CCCCGGCCCCGGCCCCGTCGAGGCCCCGGCCCCGGCCCCGGCCCCGGCCCCGGC
CCCGGCCCCGGCCCCGGCCCCGTCGAGGCCCCGGCCCCGGCCCCGGCCCCGGCC
CCGGCCCCGGCCCCGGCCCCGGCCCCGGCCCCGGCCCCGGCCCCGGCCCCGGCC
CCGGCCCCGGCCCCGTCGAGGCCCCGGCCCCGGCCCCGGCCCCGGCCCCGGCCC
CGGCCCCGGCCCCGGCCCCGGCCCCGGCCCCGGCCCCGGCCCCGGCCCCGGCCC
CGGCCCCGGCCCCGGCCCCGGCCCCGGCCCCGGCCCCGGCCCCGTCGAGGCCCC
GGCCCCGGCCCCGGCCCCGGCCCCGGCCCCGGCCCCGGCCCCG

– end of forward sequencing read

– end of reverse sequencing read

GGCCCCGGCCCCGGCCCCGGCCCCGGCCCCGGCCCCGGCCCCGGCCCCGGCCCC
GGCCCCGGCCCCGGCCCCGGCCCCGGCCCCGGCCCCGGCCCCGGCCCCGGCCCC
GGCCCCGGCCCCGGCCCCGGCCCCTCGAGGCCCCGGCCCCGGCCCCGGCCCCGG
CCCCGGCCCCGGCCCCGGCCCCGGCCCCGGCCCCGGCCCCGGCCCCGGCCCCGG
CCCCGGCCCCGGCCCCGGCCCCGTCGAGGCCCCGGCCCCGGCCCCGGCCCCGGC
CCCGGCCCCGGCCCCGGCCCCGGCCCCGGCCCCGGCCCCGGCCCCGTCGACTCAC
TACCTCCCTTTAAAGCCTGCTTTTTTGTACATGTAGAATTCATGATGGGCAGTGTC
GAGCTGAATCTGAGGGAGACTGAGCTGTGTCTTGGTCTTCCCGGTGGAGATACA
GTGGCTCCGGTAACCGGAAACAAGAGAGGGTTCTCAGAGACGGTTGATCTGAAG
CTAAATCTGAATAATGAGCCTGCAAACAAGGAAGGATCTACGACTCATGACGTC
GTGACTTTTGATTCCAAGGAGAAGAGTGCTTGTCCTAAAGATCCAGCCAAACCTC
CGGCCAAGGCACAAGTTGTGGGATGGCCACCGGTGAGATCATACCGGAAGAACG
TGATGGTTTCCTGCCAAAAATCAAGCGGTGGCCCGGAGGCGGCGGCGTTCGTGA
AGGTATCAATGGACGGAGCACCGTACTTGAGGAAAATCGATTTGAGGATGTATA
AAAGCTACGATGAGCTTTCTAATGCTTTGTCCAACATGTTCAGCTCTTTTACCATG
GGCAAACATGGAGGAGAAGAAGGAATGATAGACTTCATGAATGAGAGGAAATT
GATGGATTTGGTGAATAGCTGGGACTATGTTCCCTCTTATGAAGACAAAGACGGT
GATTGGATGCTCGTCGGCGACGTTCCTTGGCCAATGTTCGTCGATACATGCAAGC
GTTTACGTCTCATGAAAGGATCGGATGCCATTGGTCTCGCTCCGAGGGCGATGGA
GAAGTGCAAGAGCAGAGCTTAGCCCGGCGGCATGGACGAGCTGTACAAGTAATA
CGTAGATCCAGACATGATAAGATACATTGATGAGTTTGGACAAACCACAACTAG
AATGCAGTGAAAAAAATGCTTTATTTGTGAAATTTGTGATGCTATTGCTTTATTTG
TAACCATTATAAGCTGCAATAAACAAGTTAACAACAACAATTGCATTCATTTTAT
GTTTCAGGTTCAGGGGGAGGTGTGGGAGGTTTTTTAATTCGCGGCCAACTTTATT
ATACATAGTTGATAATTCACTGGCCGTCGTTTTACGGTACTACGGTACGGATCCT
TCAGGGGTGGATCCTTCAGGGGTGTCGCTTGGTGATTTCCAAAAATCAAATTAAT
TTTATTAAACTATTAGAACGAGCATGTTTTGTCTATATGCTACAGAAGATAAAAA
ATAATAGGAGTTAACAGTTATAAAACAACACACTTTGTTTCTATTGATTGTTGAC
CACACTGGGGTCTCATTAAGTTAGATTAAAGACACACTAACTGGGTCAAATGCA
GCAGATTGATTTCATGGCACCAGGGTAAACTTTCTAACACTTTTACGGCAATCAT
ATACATCAAAATTAAATACAGGCCACGACTGAACAAGGAGGATGATCTCCAAAA
TTAAACAAAGAGACTTGTGCCTATTTCTCTGAGGGTAAACATGACCTCTCAAGTT
AGCAAGTTGTTTTTAACACTACAAAAATAGTTAAGACTGCAATCCCAGAATAAA
GTATTGGTTTTAACCAATCAATATAGTACAGTAAACATCCATTTGTTTTGTTGAAA
CGTTAAACGAATCTGACCAAGCTATTAGCTTATATAAAACAGGTTTGCCTTTTAT
GTAGCTGAAAATACCACAGGCCCGATTTTGCTACTGTGTAAAACATTTCAGCAAG
ATTTTTTTTATATTGCATTTTTTTCTACTGAATCGTTCAAACATTTTATCATTTTAG
TTTGTTCATTCATTGCAACTGGAAAAACAACACACCACACAACCGCACATTTTTC
AGCAATAAGTACAATAAAACACTCAAATAAAAAAAAACTTTTTAAATCTCTTTGT
ATTTTTGACCGCTGTTTCGCGTAATTTCACGGTAAAACTCTGGAAATCTCCACTAC
ATTCCTCTCAGCGGCTCCTCTCAATGACAGCTGAAGAAGTGACGCGGCTGCCTGC
TGTGTTTTGATTGGTCGAATTCACTGGAGGCTTCCAGAACAGTGTAGAGTCTGAA
CGGGTGCGCGCTCTGCTGTATTTAAAGGGCGAAAGAGAGACCGCAGAGAAACTC
AACCGAAGAGAAGCGACTTGACAAAGAAGAAAAGAGCAGCCTGACAGGACTTT
TCCCCGACGAGGTGTTTATTCGCTCTATTTAAGAATCTACTGTAAGGTAAGTCTC

AATATATTGTACTCTAGTGGCTAATCAAAATTTTATAGAGATTATATGTACTTAAT
GTCAAAAAATCTACTTTGTATATGTAATCTTTTTACATGTGGACTGCCTATGTTCA
TCTTATTTTAGGTCTACTAGAAAATTATATTTCCCGTTTTCACAATAAGGATTTAA
AAAAAAAGCAATGAACAGACTGGCATTTACTTTATGTTGCTGACATTATTATATA
TGAGCATAATAACCATAAATACTAGCAAATGTCCTAAATGAATTTGTGTTAATGT
TGTCTACAAAAGAAAAGAAAATTAGCGTTTTACTTGTACAACTAATAATAACTTA
GTTATTAAGAGAATTTCACTTGTTGACTAGAAAAATCCTTTCATAATGAAACAAT
TGCACCATAAATTGTATAAATATAAAATTAATTCTAATTGTTTTTTTTTTTCCTGC
AGTCGAGCGCCACCATGGCCTACTCCGAGAAAGTCATCACCGAGTTCATGCGCTT
CAAGGTGCGCATGGAGGGCACCGTGAACGGCCACGAGTTCGAGATCGAGGGCG
AGGGCGAGGGCCGCCCCTACGAGGGCCACAACAACGTGAAGCTGAAGGTGACC
AAGGGCGGCCACCTGCCCTTCGCCTGGGACATCCTGTCCCACCAGTTCCAGTACG
GCTCCAAGGTGTACGTGAAGCACCCCGCCGACATCACCGACTACAAGAAGCTGT
CATTCCCCGAGGGCTTCAAGTGGGAGCGCGTGATGAACTTCGAGGACGGCGGCG
TGGCGACAGTGACCCAGGACTCCTCCCTGCAGGACGGCTGCTTCATCTACAAGGT
GAAGTTCATCGGAGTGAACTTCACCTCCGACGGCCCCGTGATGCAGAAGAAGAC
CATGGGCTGGGAGGCCTCAACCGAGCGCATGTACCCCCGCGACGGCGTGCTGAA
GGGCGAGACCCACAAGGCCCTGAAGCTGAAGGACGGCGGCCACTACCTGGTGGA
GTTCAAGTCCATCTACATGGACAAGAAGCCAGTGCAGCTGACCGGCTACTACTA
CGTGGACGCCAAGCTGGACATCACCTCCCACAACGAGGACTACACCATCGTGGA
GCAGTACGAGCGCACCGAGGGCCGCCACCACCTGTTCTAAGCGGCCGCGACTCT
AGATCATAATCAGCCATACCACATTTGTAGAGGTTTTACTTGCTTTAAAAAACCT
CCCACACCTCCCCCTGAACCTGAAACATAAAATGAATGCAATTGTTGTTGTTAAC
TTGTTTATTGCAGCTTATAATGGTTACAAATAAAGCAATAGCATCACAAATTTCA
CAAATAAAGCATTTTTTTCACTGCATTCTAGTTGTGGTTTGTCCAAACTCATCAAT
GTATCTTAGGTACCTAGGGATAA
