# Supplementary figures and images for: Stable transgenic C9orf72 zebrafish model key aspects of the ALS/FTD phenotype and reveal novel pathological features
Source: Acta Neuropathol Commun. 2018 Nov 19;6:125. doi: 10.1186/s40478-018-0629-7 (PMC6240957; doi:10.1186/s40478-018-0629-7)

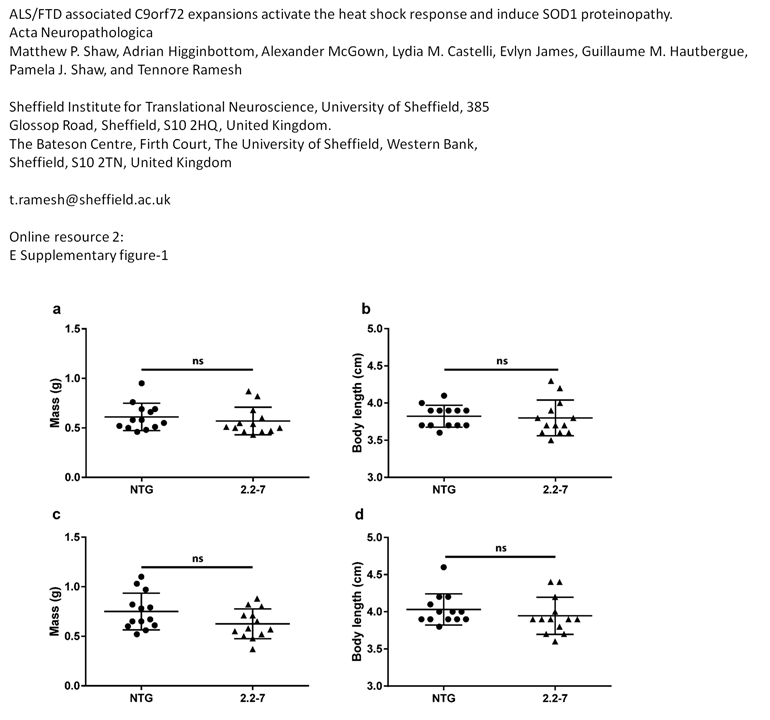

Supplement: Supplementary file 3 — Figure S1. Body mass and body length were not significantly different at the time of swim tunnel testing. (a) At 9 months old, body mass of the 2.2–7 and NTG zebrafish tested in the swim tunnel was not significantly different. N = 12 zebrafish per genotype. (b) At 9 months old, body length of the 2.2–7 and NTG zebrafish tested in the swim tunnel was not significantly different. N = 12 zebrafish per genotype. (c) At 12 months old, body mass of the 2.2–7 and NTG zebrafish tested in the swim tunnel was not significantly different. N = 13 zebrafish per genotype. (d) At 12 months old, body length of the 2.2–7 and NTG zebrafish tested in the swim tunnel was not significantly different. N = 13 zebrafish per genotype. All measurements were carried out ~ 40 min after removal from the swim tunnel (5 min rest, 30 min spontaneous behaviour recording and another 5 min of rest). (TIF 360 kb) [file 40478_2018_629_MOESM2_ESM.tif]

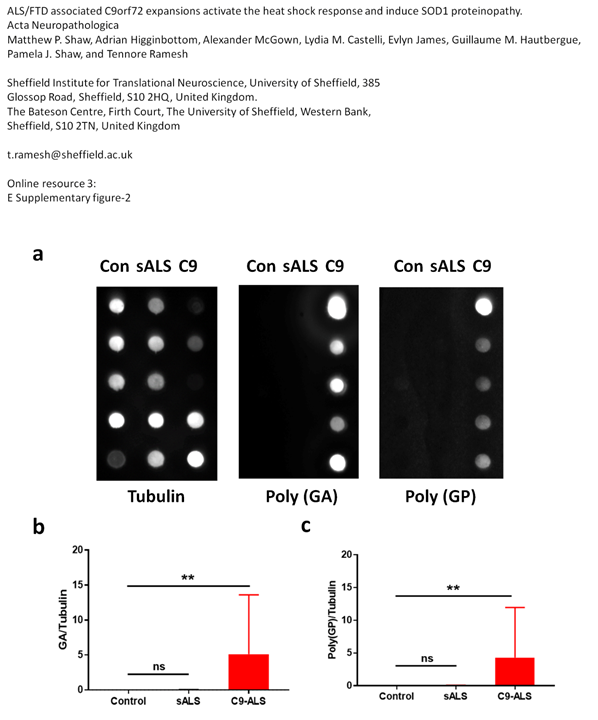

Supplement: Supplementary file 4 — Figure S2. Poly(GA) and poly(GP) DPR proteins are produced in cerebellum of C9orf72 patients. (a) Dot blots of grey matter cerebellum samples from n = 5 control, sALS and C9orf72 patients each. Immunoblotting with an antibody against tubulin reveals mostly even loading amongst the numerous samples. Immunoblotting with an antibody against poly(GA) reveals that C9orf72 patients express abundant poly(GA) DPRs, whereas control and sALS samples do not. And immunoblotting with an antibody against poly(GP) reveals that C9orf72 patients express abundant poly(GP) DPRs, whereas control and sALS samples do not. (b) Quantification showing that in cerebellum grey matter, significantly higher poly(GA) signal is detected in C9-ALS samples in comparison to control samples, when normalised to tubulin. (c) Quantification showing that in cerebellum grey matter, significantly higher poly(GP) signal is detected in C9-ALS samples in comparison to control samples, when normalised to tubulin. Con: Control, sALS: sporadic-ALS, C9: C9orf72-ALS. (TIF 485 kb) [file 40478_2018_629_MOESM4_ESM.tif]
